# Fresh Water Cyanobacteria *Geitlerinema* sp. CCC728 and *Arthrospira* sp. CCC729 as an Anticancer Drug Resource

**DOI:** 10.1371/journal.pone.0136838

**Published:** 2015-09-01

**Authors:** Akanksha Srivastava, Ratnakar Tiwari, Vikas Srivastava, Tej Bali Singh, Ravi Kumar Asthana

**Affiliations:** 1 Centre of Advanced Study in Botany, Faculty of Science, Banaras Hindu University, Varanasi, 221005, India; 2 Division of statistics, Institute of Medical science, Banaras Hindu University, Varanasi, 221005, India; 3 Council Scientific and Industrial Research, Indian Institute of Toxicology Research, Lucknow, 226001, India; Yong Loo Lin School of Medicine, National University of Singapore, SINGAPORE

## Abstract

An increasing number of cancer patients worldwide, especially in third world countries, have raised concern to explore natural drug resources, such as the less explored fresh water filamentous cyanobacteria. Six strains of cyanobacteria (*Phormidium* sp. CCC727, *Geitlerinema* sp. CCC728, *Arthrospira* sp. CCC729, *Phormidium* sp. CCC731, *Phormidium* sp. CCC730, and *Leptolyngbya* sp. CCC732) were isolated (paddy fields and ponds in the Banaras Hindu University, campus) and five strains screened for anticancer potential using human colon adenocarcinoma (HT29) and human kidney adenocarcinoma (A498) cancer cell lines. *Geitlerinema* sp. CCC728 and *Arthrospira* sp. CCC729 were the most potent as determined by examination of morphological features and by inhibition of growth by graded concentrations of crude extracts and thin-layer chromatography (TLC) eluates. Cell cycle analysis and multiplex assays using cancer biomarkers also confirmed *Geitlerinema* sp. CCC728 and *Arthrospira* sp. CCC729 as cancer drug resources. Apoptotic studies in the cells of A498 (cancer) and MCF-10A (normal human epithelial) exposed to crude extracts and TLC fractions revealed no significant impact on MCF-10A cells emphasizing its importance in the development of anticancer drug. Identification of biomolecules from these extracts are in progress.

## Introduction

More than 60% of the world’s total cancer cases (14 million new cases per year) occur in Africa, Asia, and Central and South America, and these regions account for ~70% of cancer deaths in the world (13 million per year) [1]. A number of chemotherapeutic agents (60% of the approved drugs) for cancer are sourced from natural compounds [2]. da Rocha *et al*. have reviewed the importance of natural drug resources, including microbes [3]. Cyanobacteria, the photosynthetic oxygen-evolving prokaryotes that originated ~2.8 billion years ago [4], faced many stresses during evolution and thus, seem to have been endowed with the ability to synthesize a huge diversity of antimicrobial, anticancer, and anti-inflammatory agents [5–11]. Recently, green carbon nanotags (G-Tags) from harmful cyanobacteria have been synthesized for use in cancer therapy because of their high solubility, excellent photostability, low cytotoxicity, and ability to induce death in multiple cancer cell lines, including human hepatocellular liver carcinoma (Hep-G2) and human breast adenocarcinoma (MCF-7) [12]. Due to increase in the number of cancer patients and limitations of available drugs, problems with drug safety, narrow spectrum of activity and effectiveness, the development of newer broad-spectrum anticancer molecules from the less explored freshwater cyanobacteria is desirable.

Some earlier studies have shown that filamentous marine cyanobacteria are important sources of biomolecules for preventing cancer proliferation, neurodegenerative disorders and infectious diseases [8]. Noticeably, marine cyanobacteria produce a wide range of compounds with pro-apoptotic properties [13]. Cell shrinkage, membrane budding, and apoptosis were observed in human promyelocytic leukemia (HL-60) cells exposed to the aqueous extracts of unicellular marine strains of *Synechocystis* and *Synechococcus* [14]. Therefore we intend to screen fresh water cyanobacteria for anticancer properties on selected cell lines.

In the present communication, we have screened five freshwater, non-heterocystous, filamentous cyanobacterial strains (*Phormidium* sp. CCC727, *Geitlerinema* sp. CCC728, *Arthrospira* sp. CCC729, *Phormidium* sp. CCC731 and *Leptolyngbya* sp. CCC732) for their anticancer potential by using human colon adenocarcinoma (HT29) and human kidney adenocarcinoma (A498) cancer cell lines, along with normal rat- kidney cells (NRK52E) as a control. We have used the Bio-Plex Pro human cancer biomarker panel, cell cycle analysis, and a calcein-based cell viability assay for this purpose.

## Materials and Methods

### Isolation and culture of cyanobacterial strains

Cyanobacterial strains were isolated from local freshwater habitats, such as paddy fields and ponds, at the Banaras Hindu University, Varanasi, India. Samples were washed multiple times with sterile water and cultured in BG11 medium [15]. Unialgal population of strains were procured by picking up clonal population from algal medium agar plate obtained by serially diluting the source inocula. The purity of culture was routinely checked by streaking such culture on nutrient agar plates containing 0.5% of the glucose (w/v). These should be incubated and observation made after 24h. If there is a bacterial growth, they will appear, the incubation of plates for more than 6 days, may have fungal contaminants [16]. Cyanobacteria were identified based on the morphological features described by Desikachary [17] as well as 16S *rRNA* amplification [18] and strains were deposited in Centre for conservation and Utilization of blue green algae, Indian Agriculture Research Institute, New Delhi, India. Strain no. given as *Phormidium* sp. CCC727; *Geitlerinema* sp. CCC728; *Arthrospira* sp. CCC729; *Phormidium* sp. CCC731; and *Leptolyngbya* sp. CCC732. Culture were plated on agar plate (2%) and individual cultures were grown in Erlenmeyer flasks (1 L) at 28 ± 2°C with a light intensity of 14.40 Wm^-2^ provided by a cool white fluorescent tungsten light and a light/dark cycle of 18/6 h. The culture media and glassware were sterilized at 1.0546 kg cm^-2^ (15 lb) at 121°C for 15 min. All manipulations were done aseptically under a laminar flow hood (INSTECH, New Delhi, India).

### DNA isolation and PCR amplification of 16S rRNA genes

DNA from cyanobacteria was isolated using the method designed by Sambrook and Russell [19]. Cyanobacterial cultures were centrifuged at 6,800 g for 10 min, and the pellet was broken in liquid nitrogen. The pellet was suspended and vortexed in 1 mL TE buffer (pH 8.0, 10 mM Tris HCl, 1 mM EDTA) for 15 seconds. Thereafter, 30 μL sodium dodecyl sulfate (SDS, 10% w/v) was added, followed by 5 μL proteinase-K (100 μg·mL^-1^), mixed gently, and incubated at 37°C for 2 h. Subsequently, 100 μL NaCl (5 M) was added, vortexed for 15 seconds, and incubated at 65°C after adding 80 μL cetryltrimethyl ammonium bromide (CTAB, 10% w/v). The lysate was extracted with a chloroform: isoamyl alcohol mixture (24:1), and the aqueous phase was collected after centrifugation (6,800 g for 10 min). This was followed by extraction with a Tris-saturated phenol, chloroform, and isoamyl alcohol mixture (25:24:1). The aqueous phase obtained was added with 2 μL RNAse (~30 μg·mL^-1^) and incubated at 37°C for 30 min. An equal volume of isopropanol was added to precipitate the DNA, followed by centrifugation (6,800 g for 10 min). The DNA pellet was washed twice with chilled ethanol (70%), resuspended in Milli-Q water (30 μL), and stored at 4°C. Genomic DNA was quantified with a NanoDrop spectrophotometer (NanoVue Plus, GE Health Care, Uppsala, Sweden).

The 16S *rRNA* genes of the cyanobacterial DNA were polymerase chain reaction (PCR)-amplified using the cyanobacterium specific primer set: CYA106 F-5′ CGG ACG GGT GAG TAA CGC GTG A 3′ and 781R (a)-5′ GAC TAC TGG GGT ATC TAA TCC CAT T 3′ [18]. PCR was performed in a 25 μL final volume of reaction mixture containing 100 ng/ μL DNA, 2.5 μL of 10× PCR buffer with 15 mM MgCl_2_, 0.5 μL of 2.5 pmol dNTPs, 0.75 μL of 10 pmol of each primer, and 0.05 U Taq DNA Polymerase (Bangalore Genei, Bangalore, India) in a Thermal Cycler (Eppendorf, Germany). The thermal cycling profile was as follows: 3 min initial denaturation at 94°C, followed by 35 amplification cycles each consisting of 1 min denaturation at 94°C, 1 min annealing at 59.5°C, and 2 min elongation at 72°C, with a final 7 min elongation at 72°C. The PCR product was sequenced using an ABI 3130 Genetic Analyzer, Foster City, USA.

### Phylogenetic analysis

Phylogenetic tree of amplified sequences of 16S rRNA genes from target cyanobacterial strains was constructed using MEGA5 with neighbor joining algorithm.

### Nucleotide accession numbers

Partial 16S *rRNA* gene sequences of the isolates were deposited in the GenBank database (NCBI) with the accession numbers: *Phormidium* sp. CCC727, KF 595278; *Geitlerinema* sp. CCC728, KF 595279; *Arthrospira* sp. CCC729, KF 595280; *Phormidium* sp. CCC731, KM576897; *Phormidium* sp. CCC730, KM576898 and *Leptolyngbya* sp. CCC732, KJ862054.

### Preparation of cyanobacterial crude extracts

Extraction was performed adopting 40–45 d old batch cultures according to the method reported by Doan *et al*. [20]. The harvested biomass of each cyanobacterial species was lyophilized (Christ Alpha 1–2, Germany), and 1 g dry weight was extracted twice with 60 mL followed by 40 mL methanol (100%). The extract was concentrated in a rotary vacuum evaporator (Perfit, Ambala, India) at 40°C, and dried residues were dissolved in 3 mL of dimethyl sulfoxide (DMSO) for storage at 4°C until further use.

### Thin layer chromatography (TLC) purification of crude extracts

The methanolic extracts (100 mg·mL^-1^) of the target cyanobacteria were subjected to TLC purification (TLC silica gel 60, Merck, Darmstadt, Germany) in a two-step process. The first step used carbon tetrachloride: methanol (9:1) as the mobile phase. The UV-illuminated orange bands on the plates were designated as A, B, C, D, and E and were dissolved in methanol (1 mL). In the second step, each eluate was subjected to TLC purification using hexane: ethyl acetate (1:1). Only the potent designated bands of the first step were subjected to the second step. Ultimately, the bands separated in the second TLC step were monitored for anticancer potential against HT29 and A498 cancer call lines. All experiments were performed in triplicate.

### Culture of cell lines

HT29, A498, NRK52E and MCF-10A cells cells were obtained from the National Centre for Cell Science (NCCS) cell line repository (Pune, India) and were maintained in Dulbecco’s modified Eagle medium (DMEM)/F-12 (Sigma) supplemented with 10% heat-inactivated fetal bovine serum (FBS) and 1× Antibiotic-Antimycotic (GIBCO-Life Technologies, Grand Island, USA) in a 5% CO_2_ atmosphere at 37°C. Cells were used at 75–80% confluence in a 75-cm^2^ culture flask. MCF-10A was kind gift of Satyakam Patnayak IITR Lucknow and cultured in DMEM/F12 high glucose (Sigma) with supplementation of Horse serum-5% (HyClone) EGF-10 ng·mL^-1^ (Invitrogen) and Hydrocortisone 0.5 μg·mL^-1^, Cholera toxin- 100 ng·mL^-1^ as well as Insulin 10 μg·mL^-1^ (Sigma). Cell were plated at 5x10^4^ cells·mL^-1^_._

### Screening for anticancer activity

Cells were seeded in 96-well plates at 3 × 10^4^ cells·mL^-1^ and 2 × 10^5^ cells·mL^-1^ in 24-well plates. The cells were allowed to adhere for 48 h under optimum culture conditions. The cells were then treated for 72 h with 200 μg·mL^-1^, 50 μg·mL^-1^, or 12.5 μg·mL^-1^ crude extracts of *Phormidium* sp. CCC727, *Geitlerinema* sp. CCC728, *Arthrospira* sp. CCC729, *Phormidium* sp. CCC731, *Phormidium* sp. CCC730, and *Leptolyngbya* sp. CCC732, containing 1, 0.5, 0.25 and 0.0625% DMSO respectively which act as negative controls. After 72 h, the medium from each well of the 96-well plates was removed, and the wells were washed with 1× PBS (pH 7.4). Calcein (2 μM, 200 μL) was added to each well, and the plates were incubated in the dark at 37°C for 30 min. Calcein is cleaved by intracellular esterase in viable cells to generate a green fluorescent protein under UV light. Fluorescent images of the cells stained with Calcein were obtained using a fluorescent microscope (Leica DM IL Wetzlar, Germany) and the fluorescence was quantified using a fluorometer (FLUOstar Omega BMG Labtech Mornington,Victoria, Australia). Phase contrast images were obtained at different magnifications using a phase contrast microscope (NIKON Eclipse Ti-S, Nikon Instruction Inc., Chicago, Illinois, USA).

### Flow cytometry analysis

Cell cycle analysis was performed by flow cytometry using Propidium iodide (PI) staining. Treated cells were trypsinized and resuspended in FBS-supplemented DMEM/F-12 medium. Following centrifugation at 300 g for 10 min, the cell pellet was washed with 1× PBS and further resuspended and fixed in 50% ethanol. The suspension was stored overnight in the dark at 4°C. The fixed cells were harvested by centrifugation at 800 g for 10 min and resuspended in staining buffer (0.1% Triton X-100, 0.2 mg·mL^-1^ RNase A, 20 μg·mL^-1^ PI) prepared in Milli-Q water. The resuspended cells were incubated at 37°C for 45 min. The cells were then analyzed for their DNA content in an Influx C7 sorter (BD Biosciences, Franklin lakes, New Jersey, United States). Cells were gated and analyzed for doublet discrimination. A population of single cells was acquired at excitation (490 nm) and emission (630 nm) wavelengths. Data analysis was performed using BD FACS Software (BD Biosciences) to identify the percentage of cells in different phases of the cell cycle.

Apoptosis analysis in MCF-10A and A498 Cells were done by apoptosis detection kit (BD Biosciences, San Jose, CA, USA) according to the manufacturer’s protocol. Cells (5x10^4^ cells·mL^-1^) were plated on 24- well culture plate up to 24 h. These cells were exposed to C4, C5, D3 and D4 TLC fractions (12.5 μg·mL^-1^) for 72 h. These cells were harvested, washed with PBS, resuspended in 100 μL of binding buffer containing 5μL of Annexin V and Propidium Iodide (PI) and incubated for 10 min at room temperature in the dark. Subsequent to the incubation, samples were diluted by adding 400 μL of binding buffer and analyzed using BD FACS Canto II flow cytometer equipped with FACS Diva software, version 6.1.2 (BD Biosciences, San Jose, CA, USA). The results were expressed as Annexin V- FITC+ and PI- cells identified as apoptotic cells, Annexin V-FITC+ and PI+ cells as late apoptotic cells, Annexin V-FITC- and PI+ cells as necrotic cells and Annexin V-FITC- and PI–as healthy cells.

### Multiplex analysis for cancer biomarkers

Frozen supernatants of the control and treated samples were centrifuged at ≥12000 *g* for 10 min at 4°C. The Bio-Plex Pro Human Cancer Biomarker Panel 1 Assay Kit (Bio–Rad Laboratories, Hercules, CA) was used for quantification of cancer biomarkers using Bio-Plex MAGPIX Multiplex Reader (Bio-Rad Laboratories). The cancer biomarker panel quantifies the expression of 16 proteins known to have a role in progression of different human cancers using a bead-based suspension phase assay. Supernatants obtained from the samples were incubated with antibody-coated beads and then washed and incubated with biotinylated detection antibody. The beads were further washed and incubated with streptavidin-phycoerythrin (PE) conjugate. Biomarkers were quantified using PE fluorescence intensity on the MAGPIX system. The results were expressed as mean fluorescence intensity (MFI) or as pg·mL^-1^ of protein.

### Statistical analysis

To find out the significant differences in anticancer activity of crude extracts and TLC fractions, one way analysis of variance (F test) followed by Post Hoc (Student Newman Keuls) test was used to find out paiwaise significant differences between the mean values. Student t- test was used to find out the significant mean difference for variables between cancer marker proteins and crude extracts of target cyanobacteria. All statistical analysis has been performed with SPSS 16.0 trial version.

## Results

### Phylogeny of PCR amplified 16S rRNA of target cyanobacterial strains

**Fig 1:**
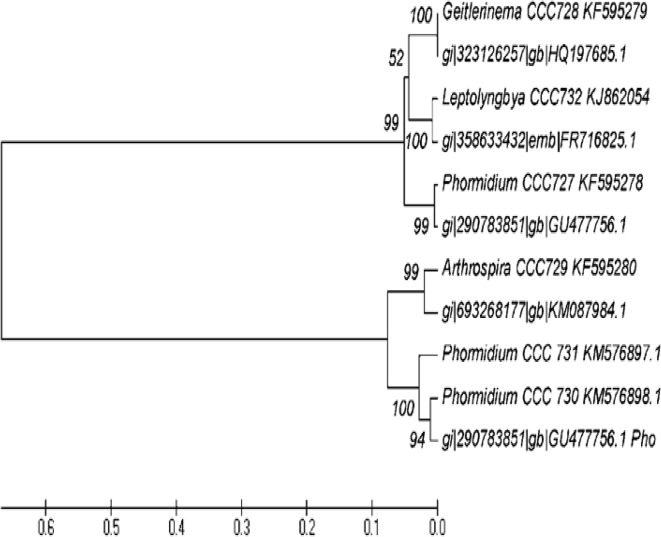
Phylogenetic tree of 16S rRNA gene in cyanobacteria generated through BLASTN search in NCBI cyanobacterial database using the neighbor joining algorithm provided by MEGA 5.

The 16S rRNA genes of *Phormidium* sp. CCC727, *Geitlerinema* sp. CCC728, *Arthrospira* sp. CCC729, *Phormidium* sp. CCC730, *Phormidium* sp. CCC731 and *Leptolyngbya* sp. CCC732 were amplified. These amplified sequences were further compared with the reported organisms available in NCBI database, and were found to be closely related as represented in S1 Table, showing very close identity and query coverage area with the similar genera with the different accession number, confirmed our findings. Phylogenetic relationship clearly demonstrated that *Arthrospira* sp. CCC729, *Phormidium* sp. CCC730 and *Phormidium* sp. CCC731 in the different cluster, while *Phormidium* sp. CCC727, *Geitlerinema* sp. CCC728, *Leptolyngbya* sp. CCC732 clustered together (Fig 1). Therefore we have selected only one *Phormidium* sp. CCC731 from one cluster and another *Phormidium* sp. CCC727 from different cluster (Fig 1.).

### Anticancer activity of crude extracts and TLC fractions

Anticancer activity was measured by the calcein assay. Crude DMSO extracts (12.5, 50, and 200 μg·mL^-1^) of cyanobacteria *Phormidium* sp. CCC727, *Arthrospira* sp. CCC729, *Geitlerinema* sp. CCC728, *Leptolyngbya* sp. CCC732, and *Phormidium* sp. CCC731. These measures were tested for their anticancer potential against HT29 and A498 cells (see S1–S11 Figs). The sensitivity of HT29 cells against the crude extract of *Arthrospira* sp. CCC729 showed that treatment with 200 μg·mL^-1^ completely inhibited growth (S3 Fig). HT29 cells seemed to have disaggregated with increased concentrations of crude extracts, and deformities in the cells were noted with treatment at 200 μg·mL^-1^. The crude extract of *Geitlerinema* sp. CCC728 appeared to be more potent than that of *Arthrospira* sp. CCC729 (S4 Fig). The crude extracts of *Phormidium* sp. CCC727 (S5 Fig), *Leptolyngbya* sp. CCC732 (S6 Fig), and *Phormidium* sp. CCC731 (S2 Fig) could not inhibit the growth of HT29 cells even at 200 μg·mL^-1^.

**Fig 2:**
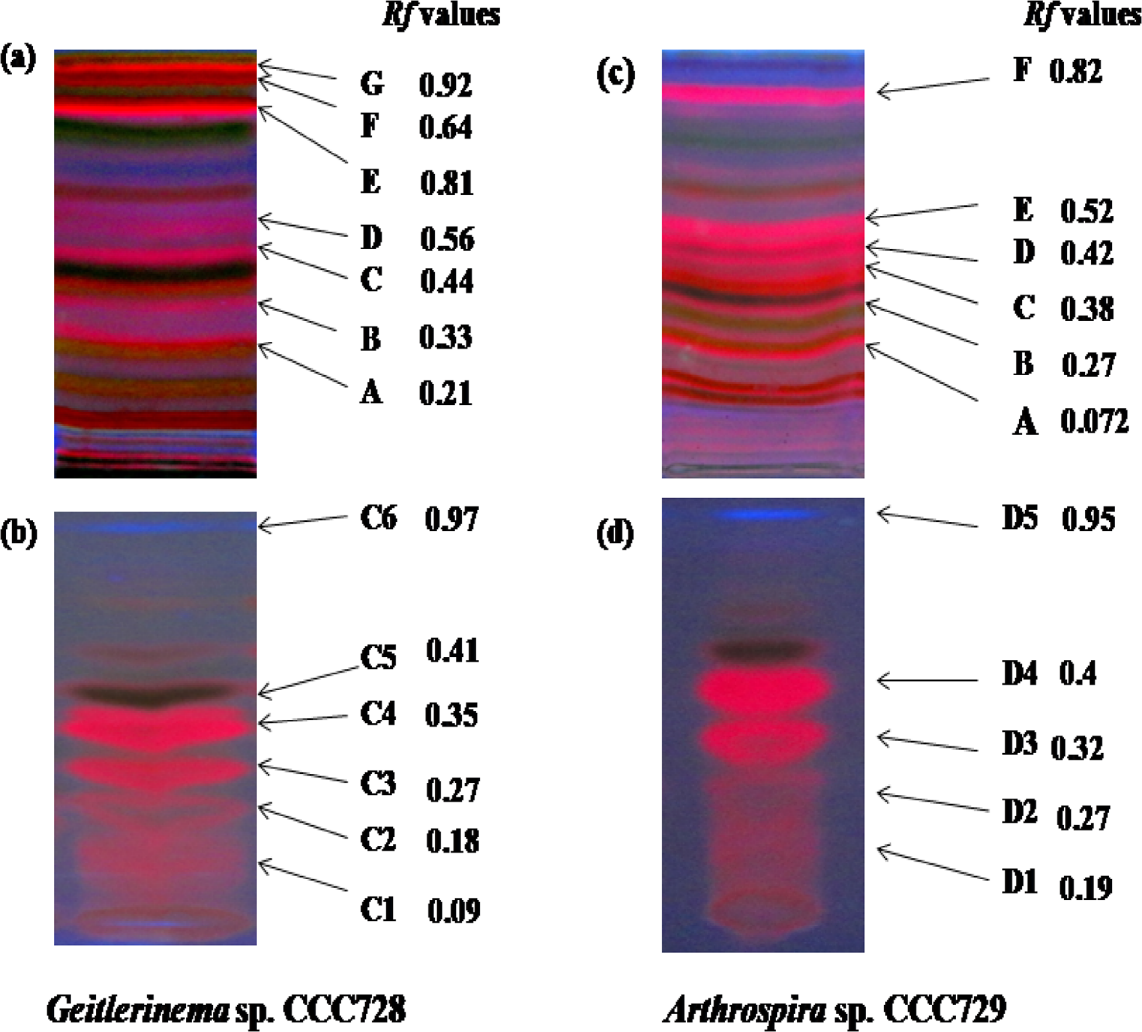
TLC pattern of cyanobacterial extracts. TLC patterns of methanolic extracts of lyophilized *Geitlerinema* sp. CCC728 **(a)** and *Arthrospira* sp. CCC729 **(c),** using CCl4: methanol (9:1). The most potent bands from *Geitlerinema* sp. CCC728 **(b)** and *Arthrospira* sp. CCC729 (d), were purified by a second TLC step using hexane: ethyl acetate (1:1).

Based on this preliminary screening for anticancer activity, we selected *Geitlerinema* sp. CCC728 and *Arthrospira* sp. CCC729 for further screening. The crude extracts were purified by a two-step TLC as described. The designated bands (A, B, C, D, E, and F) from the first TLC (Fig 2A and 2C) purification of cyanobacterial crude extracts were screened against A498 cells (S12–S14 Figs). Fraction C of *Geitlerinema* sp. CCC728 (*Rf* = 0.44) and fraction D of *Arthrospira* sp. CCC729 (*Rf* = 0.42) were selected for further purification by a second TLC step (Fig 2B and 2D).

**Fig 3:**
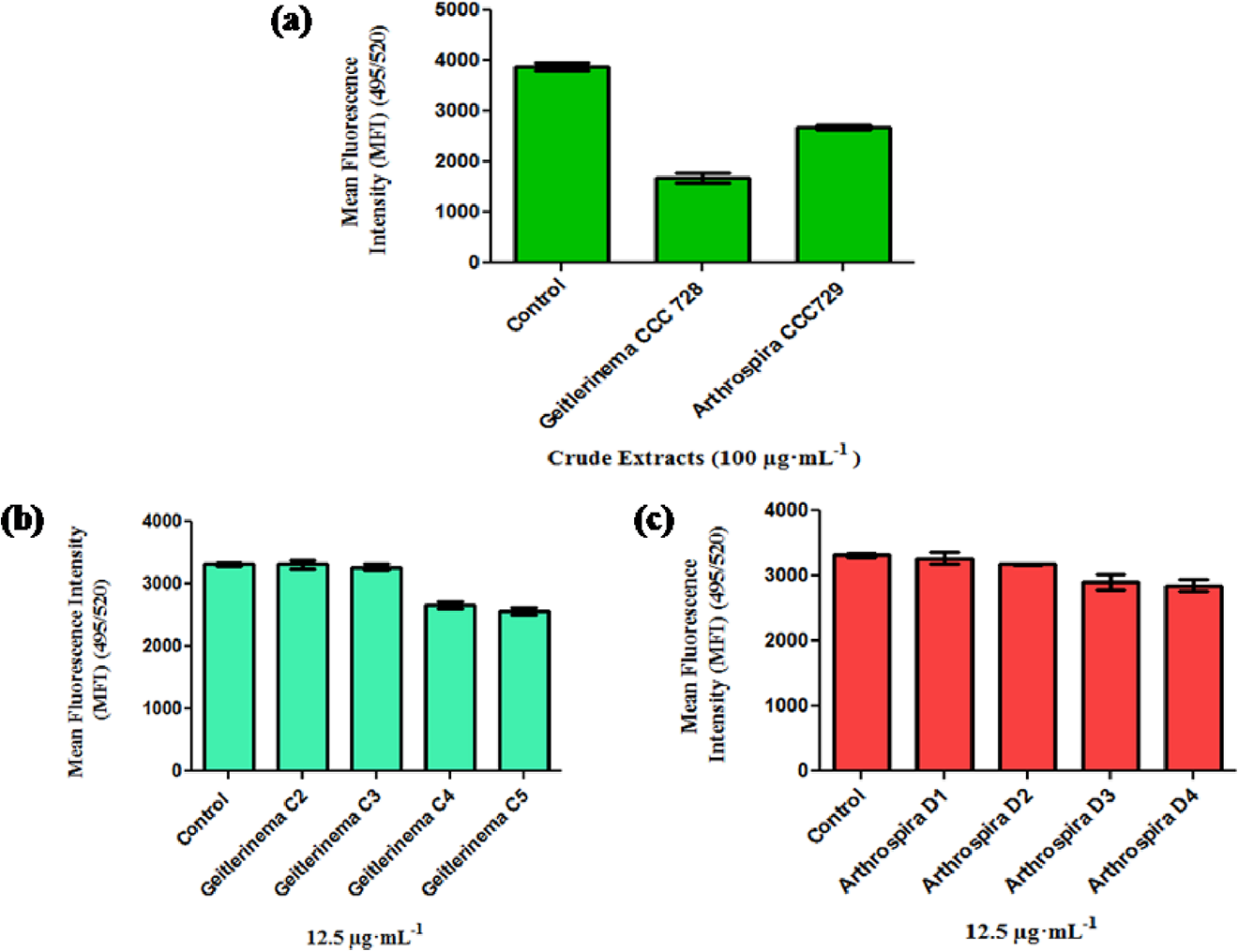
Screening for anticancer activity on cancer cell line. Calcein assay using a renal cell carcinoma (A498) cell line. Significant difference (p<0.001) between pairs, control with *Geitlerinema* CCC728 and *Arthrospira* CCC729 **(a),** Control, C2 and C3 with C4 and C5 **(b)** and Control and D1 with D3 and D4, D2 with D4 **(c)**. Control was lacking with crude extracts or TLC fractions.

The presence of more than one UV-illuminated band indicated a mixture of compounds. The bands separated in the second step, C2, C3, C4, and C5 from *Geitlerinema* sp. CCC728 (Fig 2B) and D1, D2, D3, and D4 from *Arthrospira* sp. CCC729 (Fig 2D), were examined further for anticancer potential by using the calcein fluorescence assay. The quantitative measurement of the anticancer activity of the bands from *Geitlerinema* sp. CCC728 (C) and *Arthrospira* sp. CCC729 (D) at a low concentration (12.5 μg·mL^-1^) was compared with that of the cyanobacterial crude extracts (100 μg·mL^-1^). It is interesting to note that the crude extracts of *Geitlerinema* sp. CCC728 and *Arthrospira* sp. CCC729 showed 53% and 27.78% reduction in fluorescence measurements (Fig 3A), respectively. However, the C4 and C5 bands of *Geitlerinema* CCC728 resulted only in 19.95% and 23.07% reduction, respectively, while the C2 and C3 bands did not alter the fluorescence intensity of cancer cells compared with the control (Fig 3B). Similarly, the potent bands from *Arthrospira* sp. CCC729 (D3 and D4) at 12.5 μg·mL^-1^ showed only 12.77% and 14.87% reduction in fluorescence intensity, while the D1 and D2 eluates reduced the fluorescence intensity only marginally (average 3.19%; Fig 3C). Statistical analysis of Calcein fluorescence assay for A498 cell lines indicated that Mean Fluorescence Intensity (MFI) varied significantly due to crude extracts of *Geitlerinema* sp. CCC728 and *Arthrospira* sp. CCC729 (*F*_2,6_ = 187.896, p < 0.001), TLC fractions of *Geitlerinema* sp. CCC728 (*F*_4,15_ = 51.345, p< 0.001) and TLC fractions of *Arthrospira* sp. CCC729 (*F*_4,15_ = 7.635, p< 0.001). Post hoc SNK test revealed significant differences in MFI at p < 0.001 for crude extracts of *Geitlerinema* sp. CCC728 and *Arthrospira* sp. CCC729 (Fig 3A), TLC fractions of *Geitlerinema* sp. CCC728 (Fig 3B) and TLC fractions of *Arthrospira* sp. CCC729 (Fig 3C).

**Fig 4:**
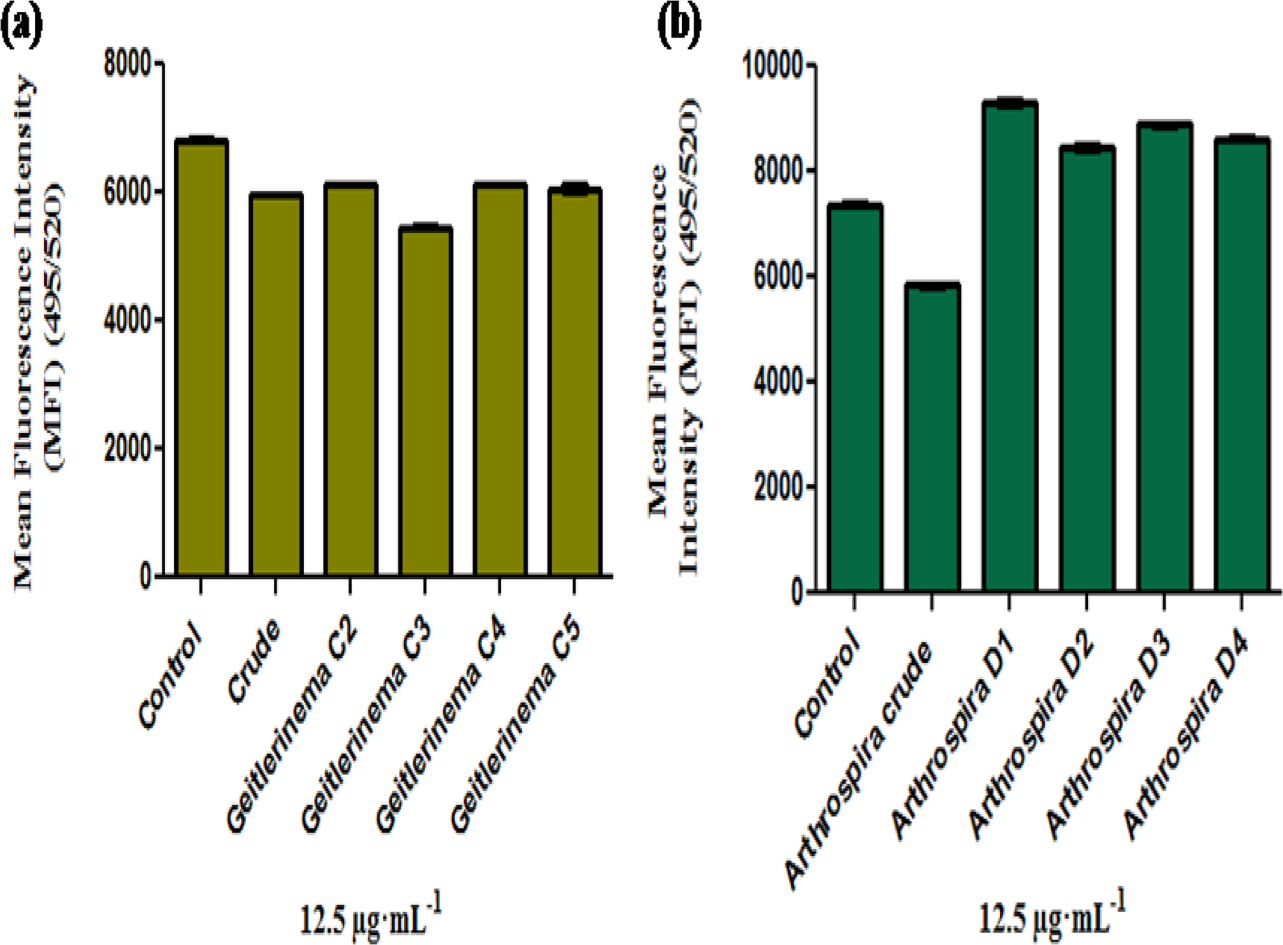
Screening for anticancer activity on NRK52E cell line. Effects of crude extracts and TLC fractions on normal rat kidney epithelial (NRK52E) cells. *Geitlerinema* sp. CCC728 crude extract and TLC fractions C2, C3, C4, and C5 (a), *Arthrospira* sp. CCC729 crude extract and TLC fractions D1, D2, D3, and D4 (b). Significant difference (p<0.001) between pairs, control with crude, C2, C3, C4 and C5, crude with control, C2, C3 and C4. C2 with control, crude and C3 **(a),** Control with crude, D1, D2, D3 and D4 **(b)**.

To confirm that these extracts have activity only in cancer cell lines, we used a normal rat kidney epithelial cell (NRK52E) and a non-tumorigenic epithelial cell line of human breast (MCF-10A). Fig 4A and 4B show the impact of the crude extract and TLC-purified fractions (C2, C3, C4 and C5) of *Geitlerinema* sp. and *Arthrospira* sp. on NRK52E cells. Both crude extracts and the TLC fractions did not show any significant activity against NRK52E cells. Lack of activity in NRK52E cells clearly indicated that these cyanobacterial strain could act as a resource of anticancer biomolecule (s) that do not significantly affect normal kidney cells. Statistical analysis of NRK52E cell lines measured by Calcein fluorescence assay indicated that Mean Fluorescence Intensity (MFI) varied significantly due to crude extract with TLC fractions of *Geitlerinema* sp. CCC728 (*F*_5,18_ = 174, p<0.001) and crude extract with TLC fractions of *Arthrospira* sp. CCC729 (*F*_5,18_ = 3.09, p< 0.001) (Fig 4A and 4B). Mean fluorescence intensity of crude and TLC fractions C2, C4 and C5 of *Geitlerinema* sp. CCC728 was 11.05% lower to that of control. C3 fraction showed 19.94% reduction in mean fluorescence intensity. Mean fluorescence intensity of crude from *Arthrospira* sp. CCC729 showed 21.49% reduction over control. Interestingly, TLC fractions D1, D2, D3 and D4 of *Arthrospira* sp. CCC729 showed increase in the mean fluorescence intensity of NRK52E cells by 19.52%. This indicated that TLC fractions of *Arthrospira* sp. CCC729 supported growth of NRK52E cells in contrast to former where C2, C3, C4 and C5 reduced mean fluorescence intensity, although at low level.

**Fig 5:**
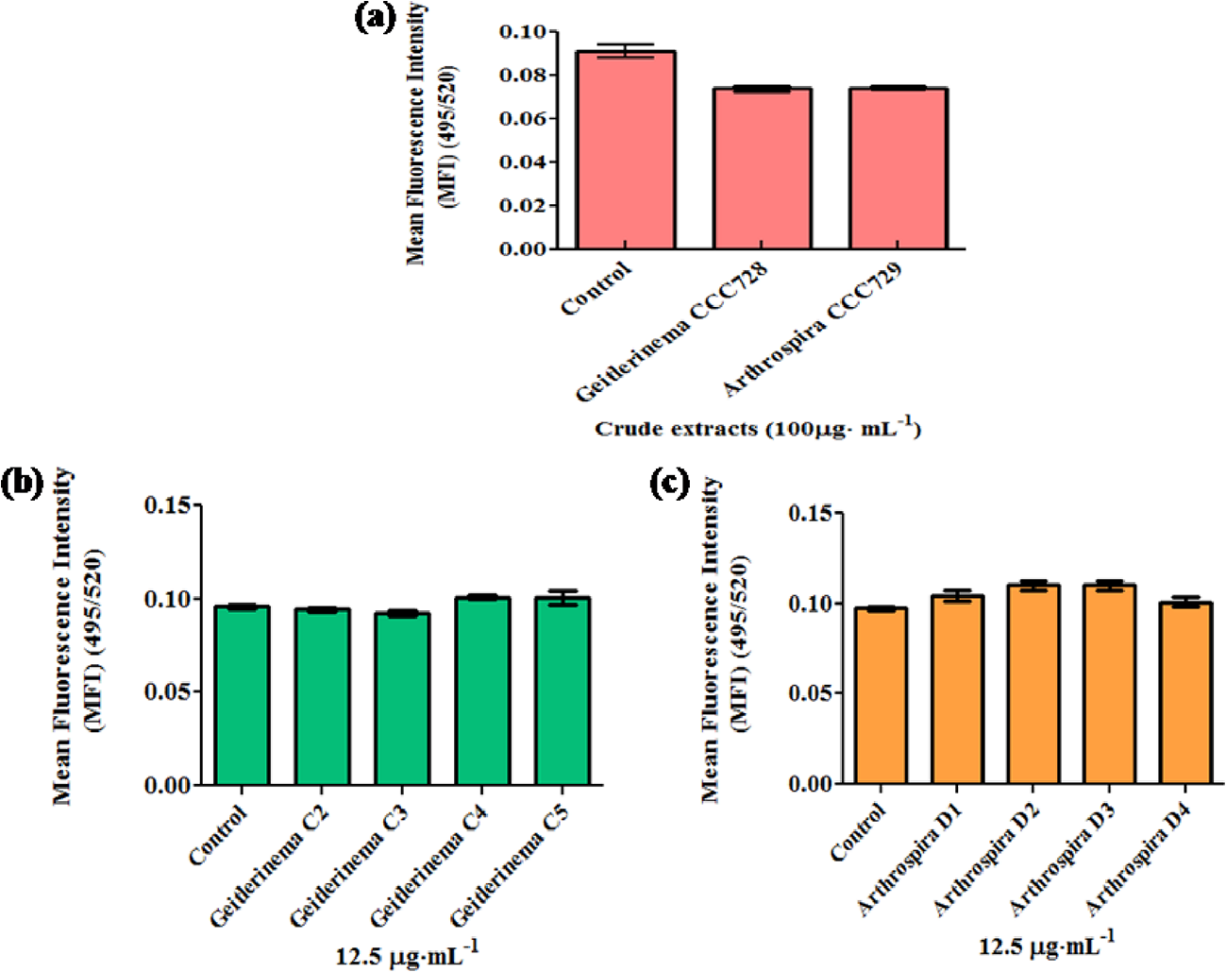
Screening for anticancer activity on MCF10A cell line. Effects of crude extracts and TLC fractions on normal human mammary epithelial (MCF10A) cells. Significant difference (p<0.001) between pairs, control with *Geitlerinema* CCC728 and *Arthrospira* CCC729 crude extracts **(a),** no significant difference (p>0.05) among pairs control and C2 and C3, C4 as well as C5 **(b)** and significant difference (p<0.05) among control with D2 with D3 and no significant difference (p>0.05) among pairs control with D1 and D4 **(c)**.

Likewise MCF-10A cells responded towards crude extracts as well as TLC purified fractions of *Geitlerinema* sp. CCC728 and *Arthrospira* sp. CCC729 (Fig 5A). Statistical analysis of MCF-10A cells lines measured by Calcein fluorescence assay indicated that Mean Fluorescence Intensity (MFI) varied significantly due to crude extracts of *Geitlerinema* sp. CCC728 and *Arthrospira* sp. CCC729 (*F*_2,6_ = 28.804, p < 0.001). TLC fractions (C2, C3, C4, and C5) from *Geitlerinema* sp. CCC728 (*F*_4,10_ = 3.841, p< 0.05) and D1, D2 D3, and D4 from *Arthrospira* sp. CCC729 (*F*_4, 10_ = 4.697, p< 0.05) also showed significant impact on MCF-10A cells lines. Post hoc (SNK) test revealed significant differences in MFI at p < 0.001 for crude extracts of *Geitlerinema* sp. CCC728 and *Arthrospira* sp. CCC729 (Fig 5A) however, no significant difference was observed among control and TLC fractions of *Geitlerinema* sp. CCC728 (p> 0.05) (Fig 5B). The significant differences in MFI at p< 0.05 among control with TLC fractions D2 and D3 of *Arthrospira* sp. CCC729 whereas no significant differences among control with D1 and D4 (p> 0.05) were obtained (Fig 5C).

### Effect of extracts on cell cycle progression

**Fig 6:**
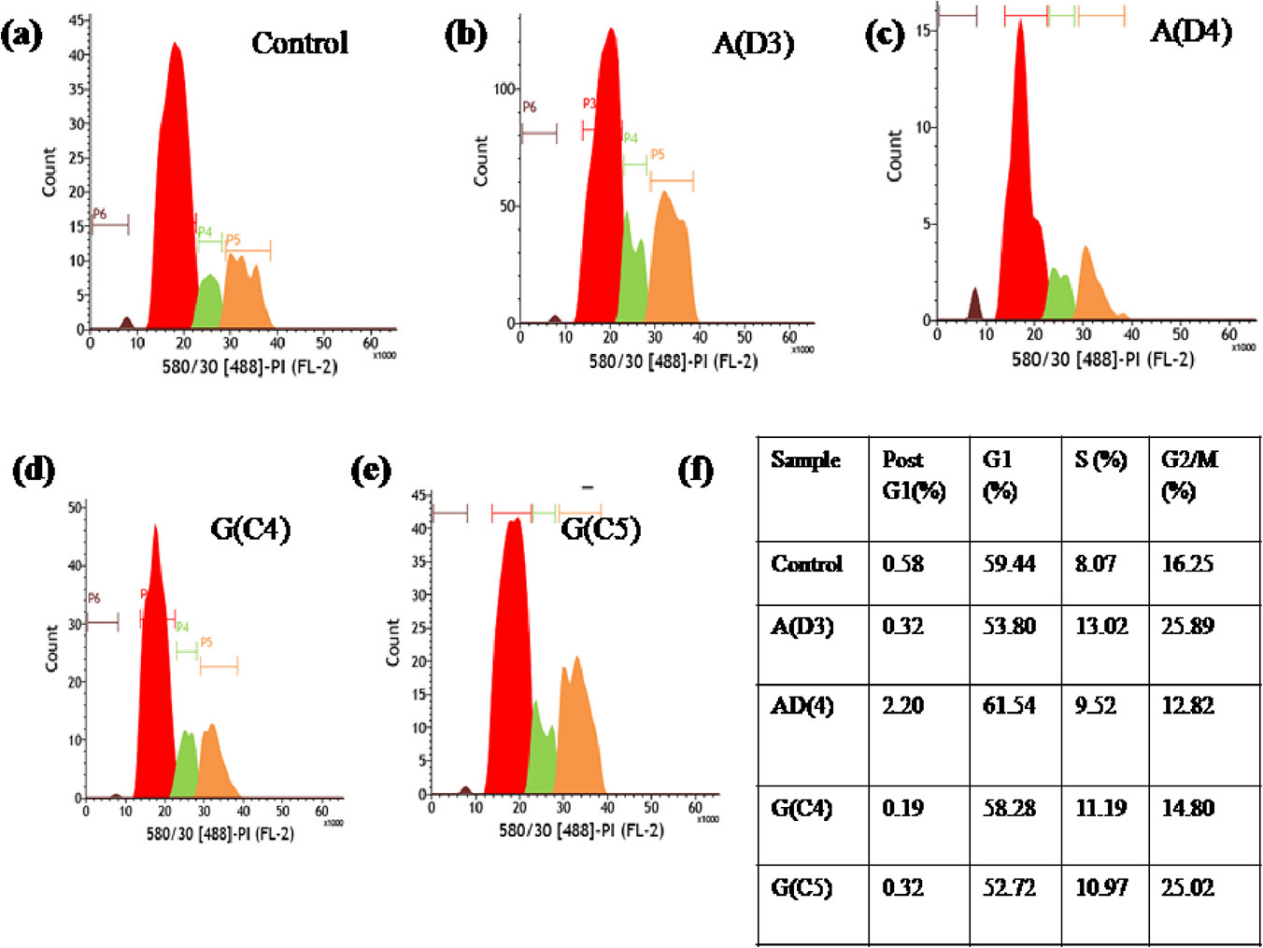
Cell cycle analysis of A498 cell lines with PI staining using Flow cytometry. Interaction of cells with most potent fractions from the second TLC purification: control cells without treatment (a), cells *vs* D3 **(b)** and D4 **(c) from** *Arthrospira*, C4 **(c)** and C5 **(d)** from *Geitlerinema*.

Cell cycle analysis was performed on two fractions of *Geitlerinema* sp. CCC728 (C4, C5) and *Arthrospira* sp. CCC729 (D3, D4). Gated and doublet-discriminated populations were assessed for PI fluorescence intensity to quantify the amount of DNA per cell. Significant changes in the cell cycle profile were observed in all cases. For the C4 and C5 fractions of *Geitlerinema* sp. CCC728, significant cell cycle arrest was seen in the S and G2/M phases, suggesting inhibition of cell cycle progression and growth. Similar results were seen with the D3 extract of *Arthrospira* sp. CCC729. However, with the D4 extract of *Arthrospira* sp. CCC729, cells were arrested in the S phase of the cell cycle. Overall, the crude extracts showed significant levels of cancer cell inhibitory activity dependent on the perturbation of cell cycle checkpoints (Fig 6).

**Fig 7:**
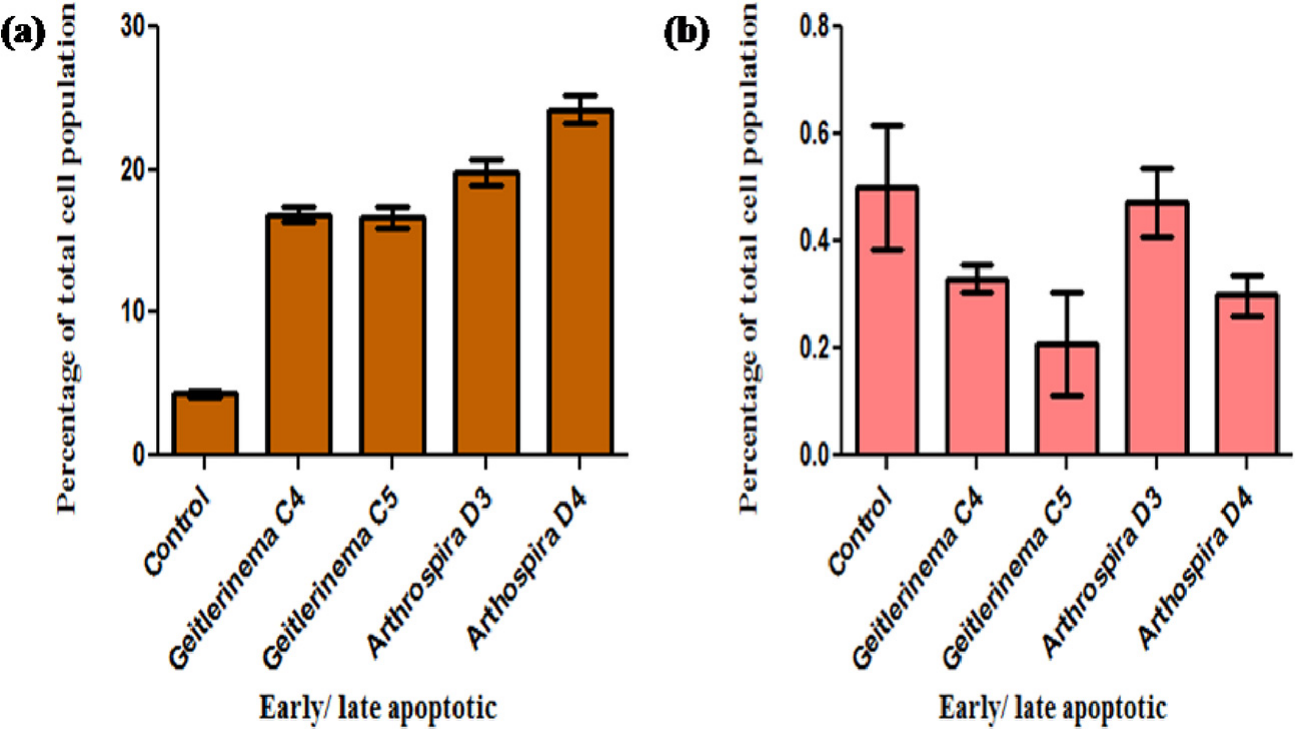
Apoptotic analysis by Flow cytometry using Annexin V-PI. A498 as well as MCF-10A cells were exposed to TLC fractions C4 and C5 (*Geitlerinema* sp. CCC728), D3 and D4 (*Arthrospira* sp. CCC729). Statistical analysis of A498 cell lines under treatment showed significant impact on cancer cells (F_4,10_ = 106.582, p<0.001) **(a).** As far as MCF-10A cells were concerned they were not significantly affected by fractions selected in experiment (F_4,10_ = 2.588, p> 0.05) **(b).**

A498 as well as MCF-10A cells were exposed to TLC fractions C4 and C5 (*Geitlerinema* sp. CCC728), D3 and D4 (*Arthrospira* sp. CCC729). The apoptotic analysis of these cells were done using annexin assay kit and data is represented in S15 and S16 Figs respectively. Statistical analysis of A498 cell lines under treatment showed significant impact on cancer cells (*F*_4, 10_ = 106.582, p<0.001) (Fig 7A). As far as MCF-10A cells were concerned they were not significantly affected by fractions selected in experiment (*F*_4, 10_ = 2.588, p> 0.05) (Fig 7B). Thus, population of A498 cells clearly undergone increased in apoptosis with exposure to C4, C5, D3 and D4 fractions in comparison to MCF-10A cells.

### Assessment of cancer biomarkers

**Fig 8:**
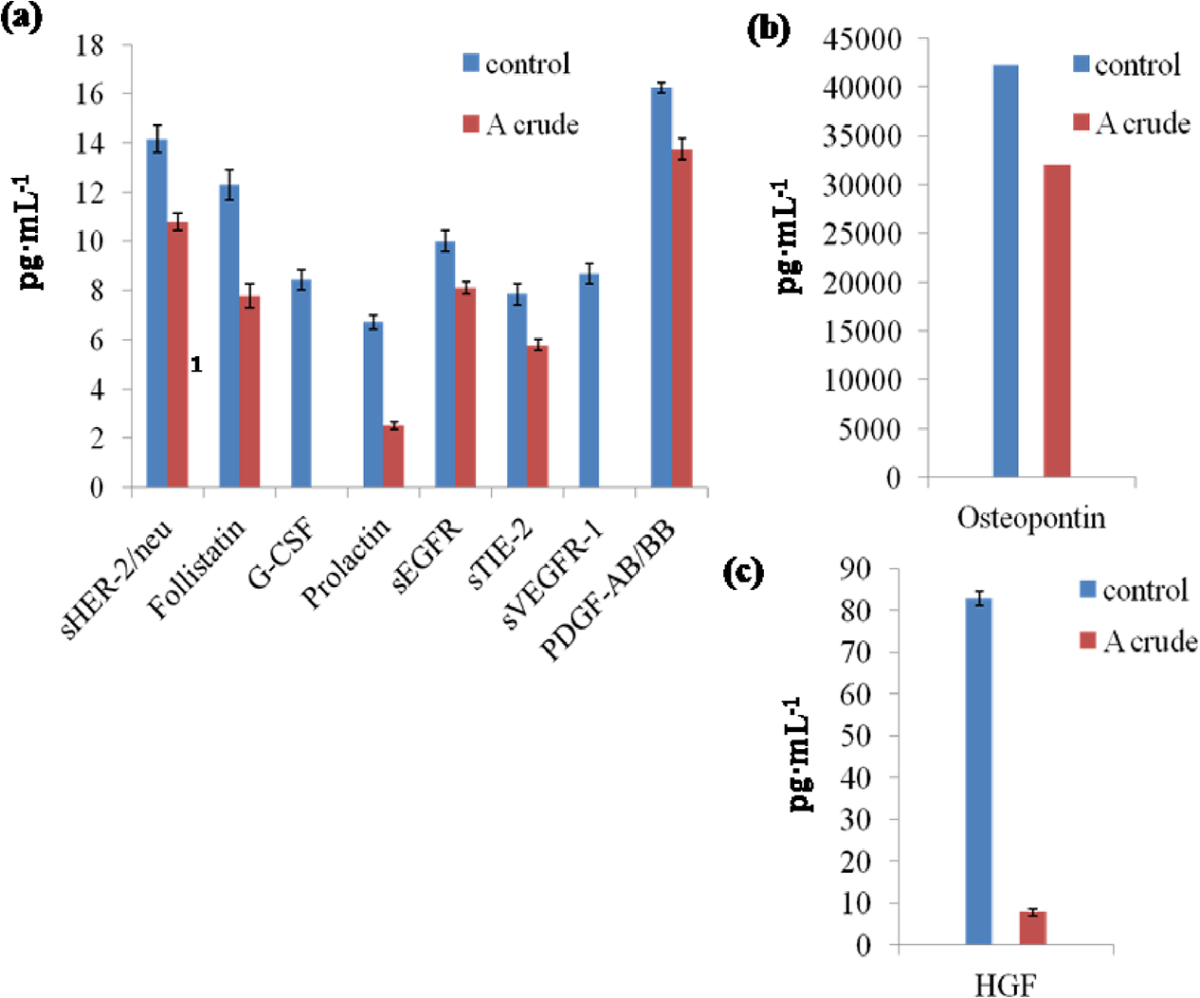
Effect of *Arthrospira* sp. CCC729 crude extract on selected cancer biomarkers. Multiplex assay showing levels of expression of cancer biomarkers in A498 cancer cells after treatment with crude extract at 100 μg·mL^-1^ in the cells.

**Fig 9:**
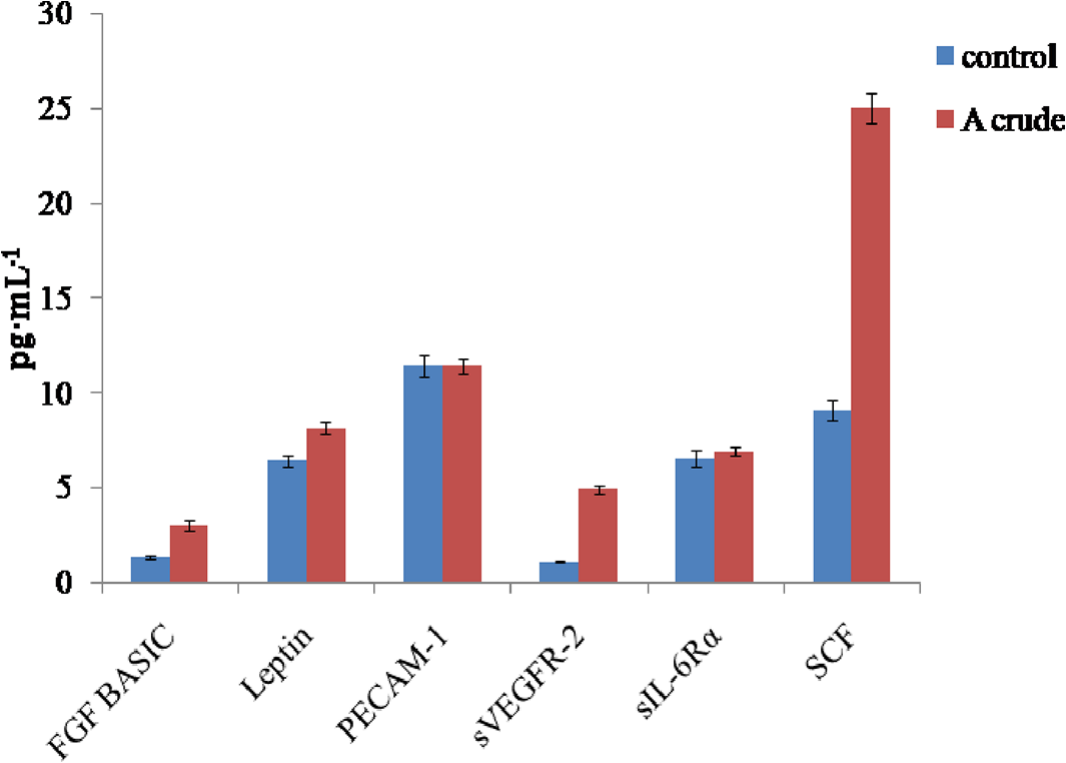
*Arthrospira* sp. CCC729 crude extract has no effect on some cancer biomarkers. Multiplex assay showing levels of expression of cancer biomarkers in A498 cancer cells after treatment with crude extract at 100 μg·mL^-1^ in the cells showing no impact on selected biomarkers.

To ascertain the anticancer potentials of *Geitlerinema* sp. CCC728 and *Arthrospira* sp. CCC729, a multiplex assay was also applied for cancer protein biomarkers. This assay was based on monitoring the levels of established cancer biomarkers in A498 cancer cells after treatment with crude extracts at 100 μg·mL^-1^. The crude extract of *Arthrospira* sp. CCC729 showed reduction in expression in the cells for ten cancer marker proteins: soluble human epidermal growth factor receptor 2 (sHER-2/neu), follistatin, granulocyte colony stimulating factor (G-CSF), prolactin, soluble epidermal growth factor receptor (sEGFR), soluble Tie2 receptor (sTIE-2), soluble vascular endothelial growth factor receptor 1 (sVEGFR-1), and platelet derived growth factor, a dimeric glycoprotein composed of two A (-AA) or two B (-BB) chains (PDGF-AB/BB; Fig 8A), as well as osteopontin (Fig 8B) and hepatocyte growth factor (HGF; Fig 8C). Of the 16 cancer marker proteins used in the multiplex assay, six proteins, basic fibroblast growth factor (FGF-basic), leptin, platelet endothelial cell adhesion molecule (PECAM-1), sVEGFR-2, soluble interleukin-6 receptor alpha (sIL-6Rα), and stem cell factor (SCF) (Fig 9), did not show reduction in expression after treatment with *Arthrospira* sp. CCC729. Multiplex analysis in the case of *Arthrospira* sp. CCC729, the “t” test indicated that the expression of crude varied significantly with their respective controls at p< 0.05 (Figs 8A, 8B, 8C and 9), except for PECAM-1 and sIL-6Rα, expression were not statistically significant (p > 0.05) (Fig 9).

**Fig 10:**
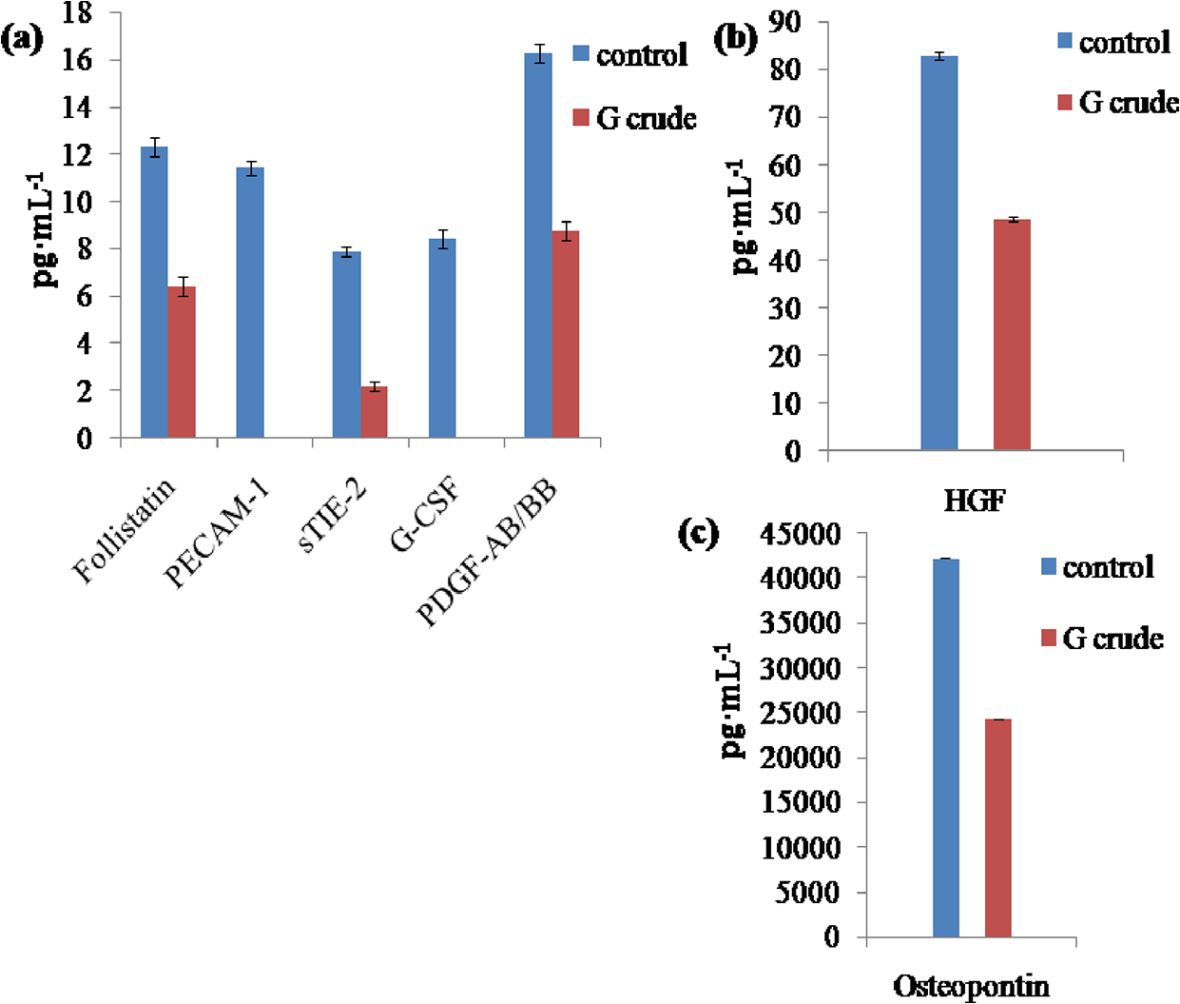
Effect of *Geitlerinema* sp. CCC728 crude extract on selected cancer biomarkers. Multiplex assay showing levels of expression of cancer biomarkers in A498 cancer cells after treatment with crude extract at 100 μg·mL^-1^ in the cells showing impact on selected biomarkers.

**Fig 11:**
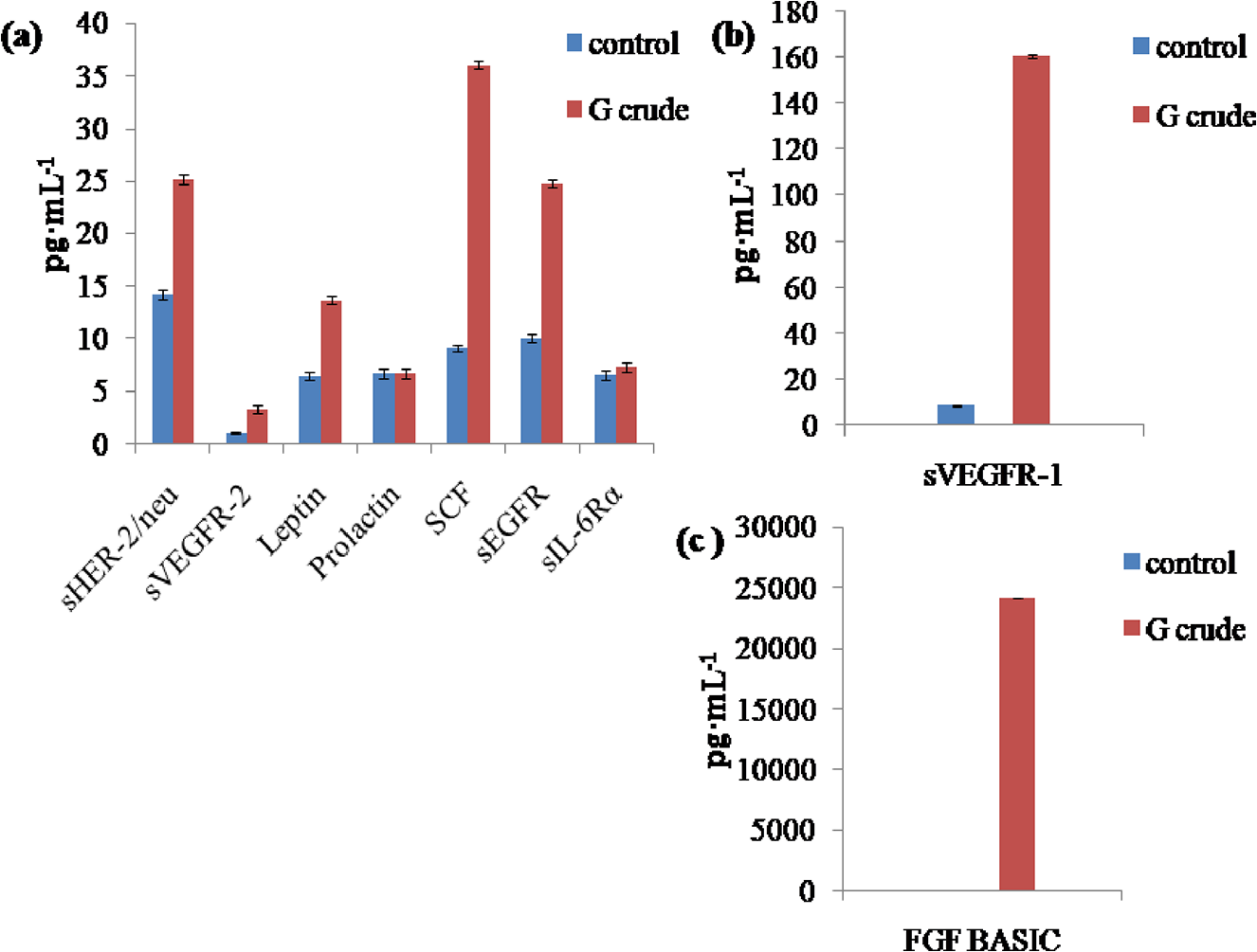
*Geitlerinema* sp. CCC728 crude extract has no effect on some cancer biomarkers. Multiplex assay showing levels of expression of cancer biomarkers in A498 cancer cells after treatment with crude extract at 100 μg·mL^-1^ in the cells showing no impact on selected biomarkers.

Likewise, the crude extract of *Geitlerinema* sp. CCC728 showed reduction in expression in cells for eight proteins: follistatin, PECAM-1, sTIE-2, G-CSF, and PDGF-AB/BB (Fig 10A), as well as HGF (Fig 10B) and osteopontin (Fig 10C). These results reiterated that *Geitlerinema* sp. CCC728 and *Arthrospira* sp. CCC729 could serve as anticancer drug resources. However, nine proteins, sHER-2/neu, sVEGFR-2, leptin, prolactin, SCF, sEGFR, sIL-6Rα, sVEGFR-1, and FGF-basic, also did not show a reduction in expression following treatment with *Geitlerinema* CCC728 (Fig 11A, 11B and 11C). Multiplex analysis in the case of *Geitlerinema* sp. CCC728, “t” test indicated that the expression of crude varied significantly with their respective controls at p< 0.05 (Figs 10A, 10B and 10C, 11B and 11C) except for Prolactin and sIL-6Rα (p = 1) (Fig 11A).

## Discussion

Phylogenetic relationship based on 16S rRNA among five different Oscillatorialean members collected from local fresh waters showed *Arthrospira* sp. CCC729 and *Geitlerinema* sp. CCC728 different clusters (Fig 1), *Geitlerinema* sp. CCC728 is in close association with *Leptolyngbya* sp. CCC732, and *Phormidium* sp. CCC727. Similar species of cyanobacteria were clustered with *Limnothrix* / *Geitlerinema–*like cyanobacterium, together in a phylogenetic study based on 16S rRNA [21]. They have also shown that *Geitlerinema* sp. AC0243 extract was toxic but unlike that of other known cyanotoxins owing probably intragenomic variations as well as 16S rRNA heterogeneity in the filamentous cyanobacterium as evident in *Leptolyngbya* sp. [22] *Arthrospira* / *Spirulina* on the other hand a healthfood and known to possess antioxidative property, rich source of carotenoids, polyunsaturated fatty acid [23; 24]. Therefore, response of crude extracts and TLC fractions of these two cyanobacteria differed towards cancer cell lines and this difference was also reflected in sensitivity towards NRK52E cells.

To date, marine filamentous cyanobacteria [13], with the exception of unicellular strains such as *Synechocystis* sp. and *Synechococcus* sp. [14], have been screened for their promising anticancer properties and active biomolecules, but the specific targets and mechanisms behind their action are not well understood. *Phromidium* sp. has been earlier shown to have anticancer activity using HT29 and HeLa cells [25]. *Oscillatoria* sp. and *Leptolyngbya* sp. were identified as sources of largamide A-H [26] and coibamide A [27], respectively. These compounds could induce apoptosis in A549 lung carcinoma, HT29 colon carcinoma cells and rat aorta 10 cells [28–29]. *Geitlerinema* sp., which is the source for ankaraholide A, shows anticancer activity against NCI-H460 lung tumor, MDA-MB-435 breast carcinoma, KB oral epidermoid and Lo Vo colon cancer cells [30]. In addition, crude extracts of selected cyanobacteria showed concentration-dependent and species-specific inhibition of growth of A498 and HT29 cancer cells. Further, the cyanobacterial crude extracts caused shrinkage in cancer cells as well as dispersion of aggregated HT29 cells but not A498 cells, indicating their response when compared with cells with DMSO only (see S1–S11 Figs). Thus, our results are in accordance with earlier studies in which cancer cells treated with anticancer agents showed typical morphological markers of apoptosis such as shrinkage and surface budding [31]. In addition to the use of morphological markers in screening for apoptotic cells, some other cellular and molecular alterations/ biomarkers associated with the death of cancer cells can also be monitored. The crude extracts of the tested cyanobacteria are mixtures of various native biomolecules; therefore, the probable anticancer biomolecules must be separated and identified for their structural identities. Based on our observations against selected cancer lines, *Geitlerinema* sp. CCC728 and *Arthrospira* sp. CCC729 proved to be potent anticancer drug sources. We adopted 12.5 μg·mL^-1^ as the concentration of the potent band eluates, compared with 100 μg·mL^-1^ of the crude extracts, in anticipation of increased effectiveness after separation from the crude materials. The purified fractions also had anticancer potential, although with lower impact (Fig 3A, 3B and 3C). Similar results were reported by Chu et al, while investigating the protective effect of aqueous extract from *Spirulina platensis* against cell death induced by free radicals and concluded that a mixture of compounds was more active than a single pure one [24]. The negligible impact on NRK52E and MCF-10A cells by purified TLC fractions emphasized the significance of such natural resources to be explored for biomolecules with anticancer activity (Figs 4 and 5). This difference in activity may be ascribed to be species specific as described earlier. Impact of crude extracts in both the cases may be attributed to presence of other than bioactive compounds.

Cell cycle analysis provided critical insights into the mechanisms of action of these extracts. Some extracts inhibited the cell cycle in the S and G2/M phases, while others inhibited the cell cycle at the G1 phase which can be one of the mechanisms for genomic instability seen in cancer cells (Fig 6). Similarly, in addition to the antiproliferative effect of *Spirulina* extract on hepatic stellate cell (HSC), the flow cytometric analysis on cell growth and cell cycle arrest revealed that aqueous extract of *Spirulina* could inhibit cell proliferation by inducing G2/M arrest in HSCs through regulating the G2 check point [24]. Likewise our data on cell cycle analysis indicated that *Geitlerinema* sp. CCC728 and *Arthrospira* sp. possess the potential for amelioration of selected cancer cells. We have also validated anticancer potential by apoptotic study, where increase in the mean percentage population of A498 cells under apoptosis was recorded after exposure to TLC purified fractions from both the cyanobacteria (Fig 7A and S15 Fig). Interestingly, none of the fractions in the investigation could induce apoptosis in MCF-10A cells (Fig 7B and S16 Fig) suggesting these cyanobacteria can act as an ideal natural anticancer resource.

*In vitro* multiplex assays yield a composite profile of clinically relevant protein biomarkers. Therefore, multiplex assays identifying different cancer biomarker proteins also confirmed the anticancer potential of *Geitlerinema* sp. CCC728 and *Arthrospira* sp. CCC729 in A498 cancer cell lines. Biomarker’s expression vary with the type of cancers. We have compiled the data in two groups, (a) group of biomarkers whose expressions were inhibited (downregulated) as Figs 8 and 10 for *Arthrospira* CCC729 and *Geitlerinema* CCC728. (b) Figs 9 and 11 are group of biomarkers whose expressions were upregulated (over expressed). Downregulation and upregulation of biomarkers might have resulted because of their interactions with certain biomolecules present in the crude extract. We observed an overall reduction in the concentration of many of these cancer related proteins following treatment of the cells with the extracts. The multiplex assays thereby verified the claim that *Geitlerinema* sp. CCC728 and *Arthrospira* sp. CCC729 are potent anticancer drug sources (Figs 8 and 10). However, some of the biomarkers showed increased expression following treatment (Figs 9 and 11). This observation indicated specificity in biomolecules interaction with protein biomarker expression. Further identification and characterization of the active biomolecules are in progress.

## Conclusions

Cancer cell lines adopted (HT29 and A498) showed deformities, cell disaggregation in morphological features after exposure to crude as well as TLC purified fractions of *Geitlerinema* sp. CCC728 and *Arthrospira* sp. CCC729. On comparison with normal rat kidney (NRK52E) and normal human epithelial (MCF-10A) cells, the crude extracts and TLC fractions of these cyanobacteria indicated a possible source of anticancer molecules. Flow cytometric analysis also showed cell cycle arrest in S and G2/M phase, suggesting of antiproliferative nature of such crude extracts and TLC fractions. Apoptotic analysis of A498 and MCF-10A cells (normal human epithelial) after exposure to TLC fractions clearly demonstrated that these fractions induced apoptosis in A498 in contrast to MCF-10A cells. Bio—plex Pro human cancer biomarker assay indicated presence of anticancer biomolecules by observing the down/ up regulation of marker proteins. These screening processes are recommended prior to isolation and identification of biomolecules for anticancer properties in little explored fresh water filamentous cyanobacteria.

## Supporting Information

S1 Fig Target cyanobacteria. (TIF)

S2 Fig Showing cell lines HT 29 (Human Colorectal Adenocarcinoma) *vs*. crude extract of *Phormidium* sp. CCC731 in DMSO. (TIF)

S3 Fig Showing cell lines HT 29 (Human Colorectal Adenocarcinoma) *vs*. crude extract of *Arthrospira* sp. CCC729 in DMSO. (TIF)

S4 Fig Showing cell lines HT 29 (Human Colorectal Adenocarcinoma) *vs*. crude extract of *Geitlerinema* sp. CCC728 in DMSO. (TIF)

S5 Fig Showing cell lines HT 29 (Human Colorectal Adenocarcinoma) *vs*. crude extract of *Phormidium* sp. CCC727 in DMSO. (TIF)

S6 Fig Showing cell lines HT 29 (Human Colorectal Adenocarcinoma) *vs*. crude extract of *Leptolyngbya* sp. CCC732 in DMSO. (TIF)

S7 Fig Showing cell lines A498 (Renal cell carcinoma (RCC) *vs*. crude extract of *Arthrospira* sp. CCC729 in DMSO. (TIF)

S8 Fig Showing cell lines A498 (Renal cell carcinoma (RCC) *vs*. crude extract of *Geitlerinema* sp. CCC728 in DMSO. (TIF)

S9 Fig Showing cell lines A498 (Renal cell carcinoma (RCC) *vs*. crude extract of *Phormidium* sp.CCC727 in DMSO. (TIF)

S10 Fig Showing cell lines A498 (Renal cell carcinoma (RCC) *vs*. crude extract of *Leptolyngbya* sp CCC732 in DMSO. (TIF)

S11 Fig Showing cell lines A498 (Renal cell carcinoma (RCC) *vs*. crude extract of *Phormidium* sp.CCC731 in DMSO. (TIF)

S12 Fig Showing cell lines A498 (Renal cell carcinoma (RCC) *vs*. TLC (using CCl4: methanol (9:1) fraction C of *Geitlerinema* CCC728 in DMSO. (TIF)

S13 Fig Showing cell lines A498 (Renal cell carcinoma (RCC) *vs*. TLC using CCl4:methanol (9:1) fraction D of *Arthrospira* CCC728 in DMSO. (TIF)

S14 Fig Showing cell lines A498 (Renal cell carcinoma (RCC) *vs*. TLC using CCl4:methanol (9:1) fraction D of *Phormidium* CCC727 in DMSO. (TIF)

S15 Fig Apoptotic analysis of A498 A cells after exposure to selected TLC purified fractions of *Geitlerinema* (C4 and C5) and *Arthrospira* (D3 and D4). (TIF)

S16 Fig Apoptotic analysis of MCF-10 A cells after exposure to selected TLC purified fractions of *Geitlerinema* (C4 and C5) and *Arthrospira* (D3 and D4). (TIF)

S1 Table A comparison of *16S* r RNA amplified sequences from target cyanobacteria with homologous organism available in NCBI database. (TIF)
