# Supplementary figures and images for: Fresh Water Cyanobacteria Geitlerinema sp. CCC728 and Arthrospira sp. CCC729 as an Anticancer Drug Resource
Source: PLoS One. 2015 Sep 1;10(9):e0136838. doi: 10.1371/journal.pone.0136838 (PMC4567175; doi:10.1371/journal.pone.0136838)

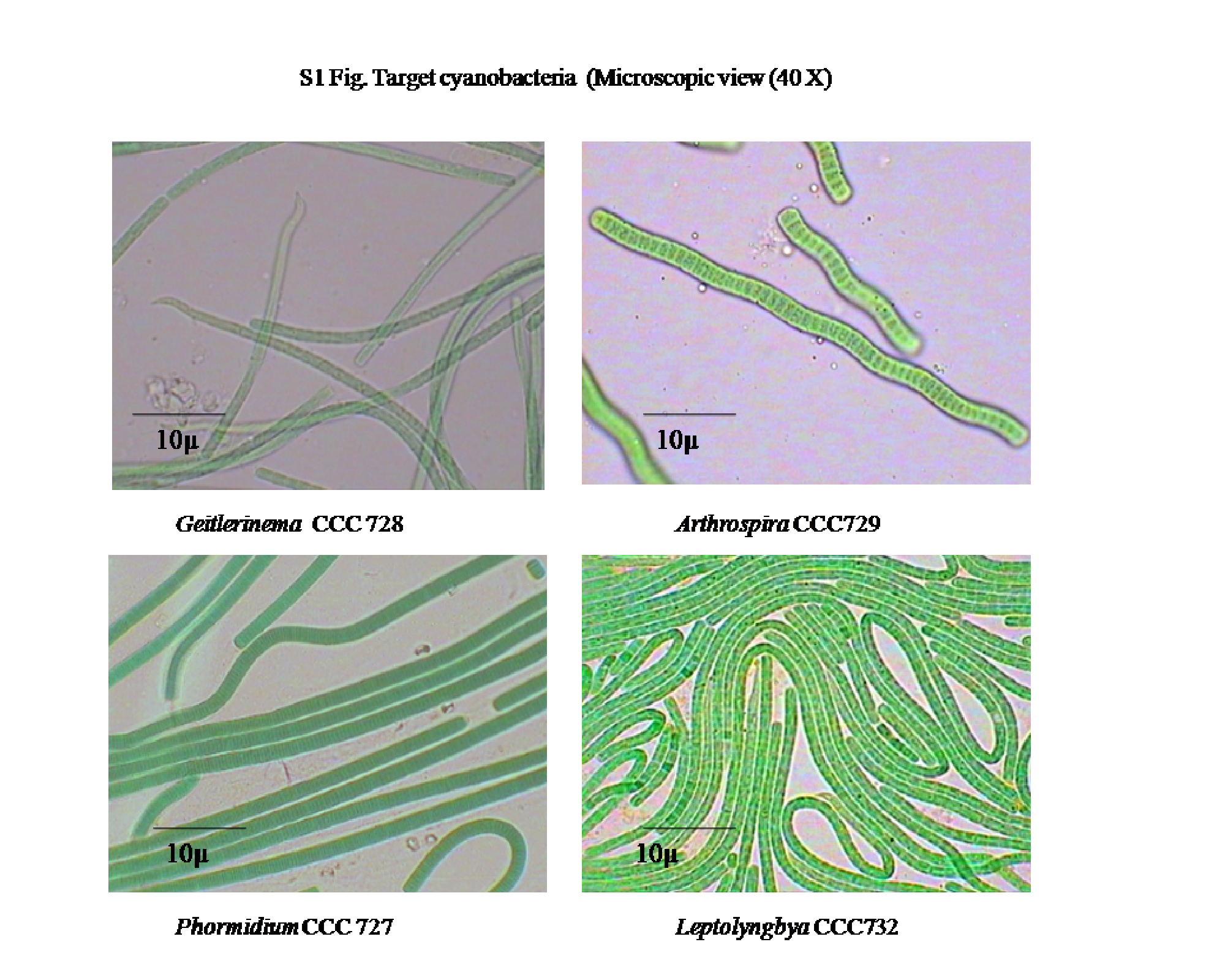

Supplement: S1 Fig — (TIF) [file pone.0136838.s001.tif]

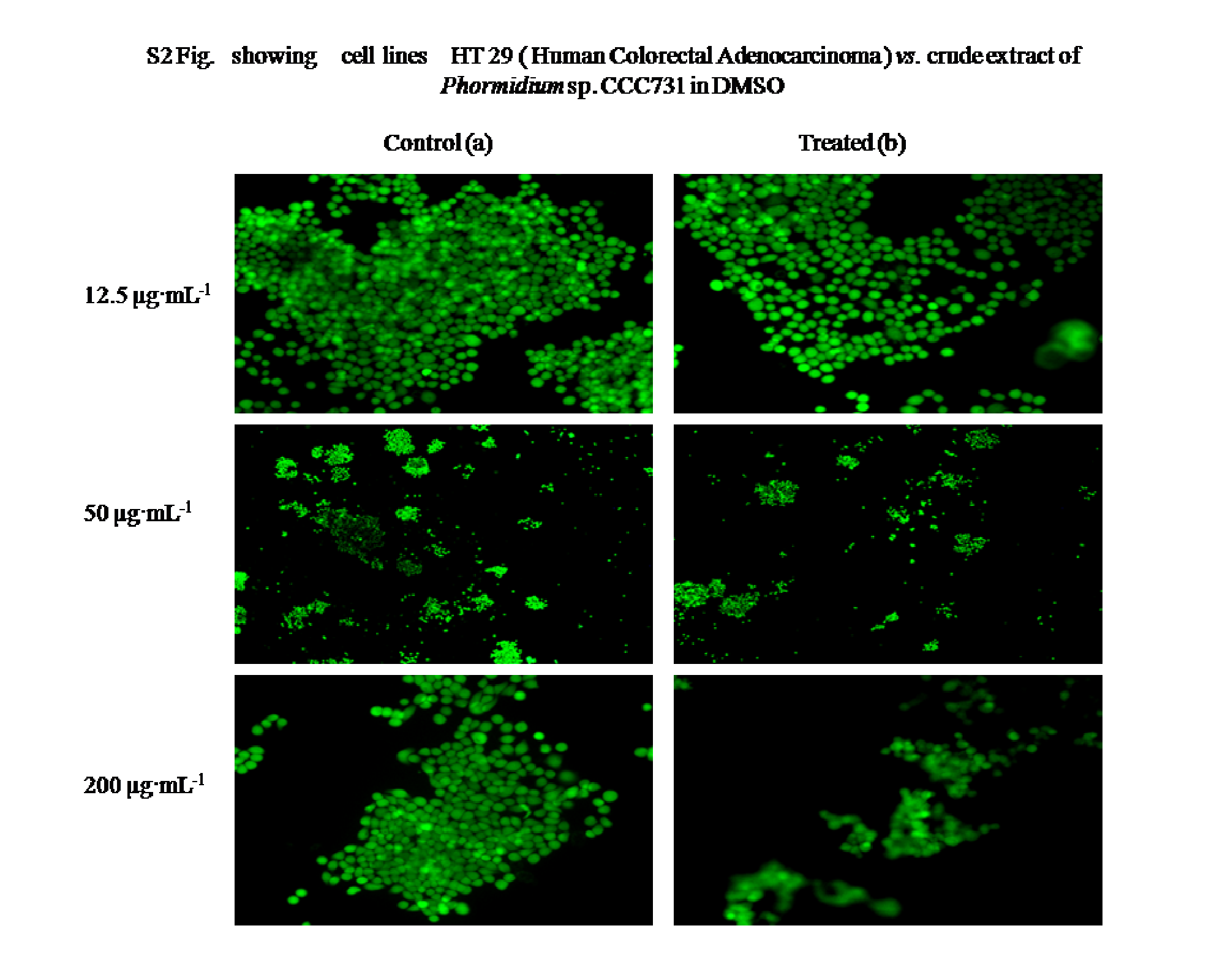

Supplement: S2 Fig — (TIF) [file pone.0136838.s002.tif]

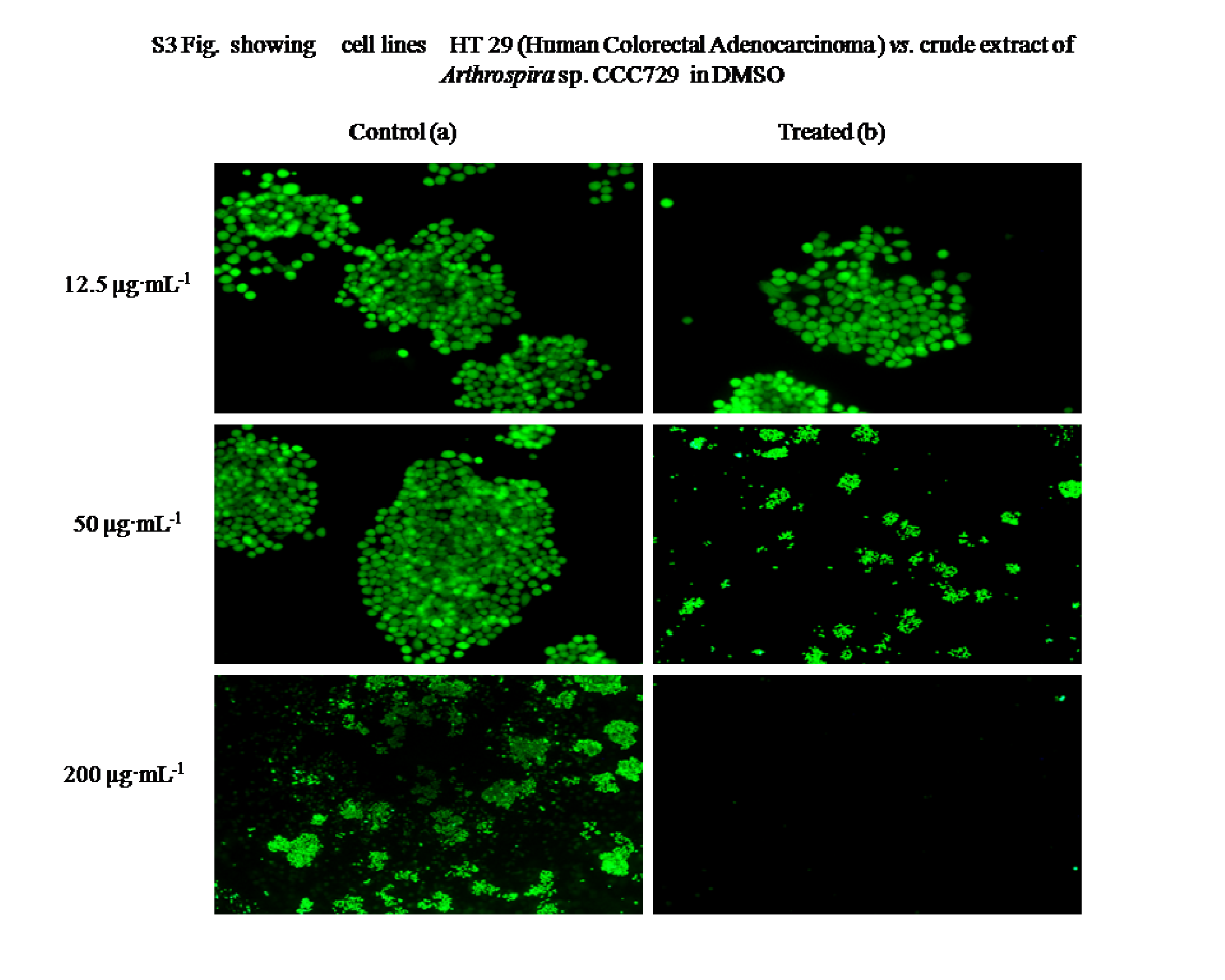

Supplement: S3 Fig — (TIF) [file pone.0136838.s003.tif]

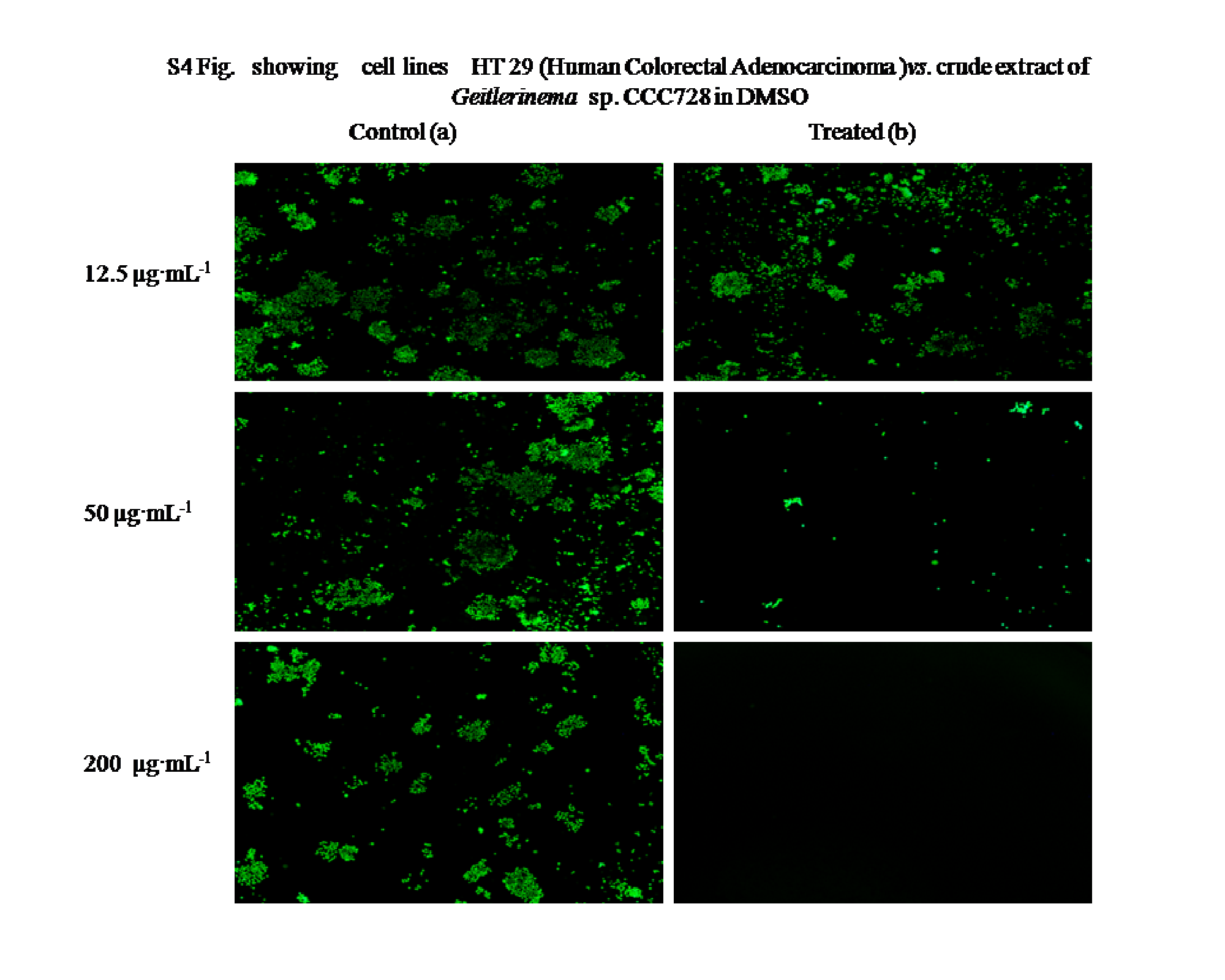

Supplement: S4 Fig — (TIF) [file pone.0136838.s004.tif]

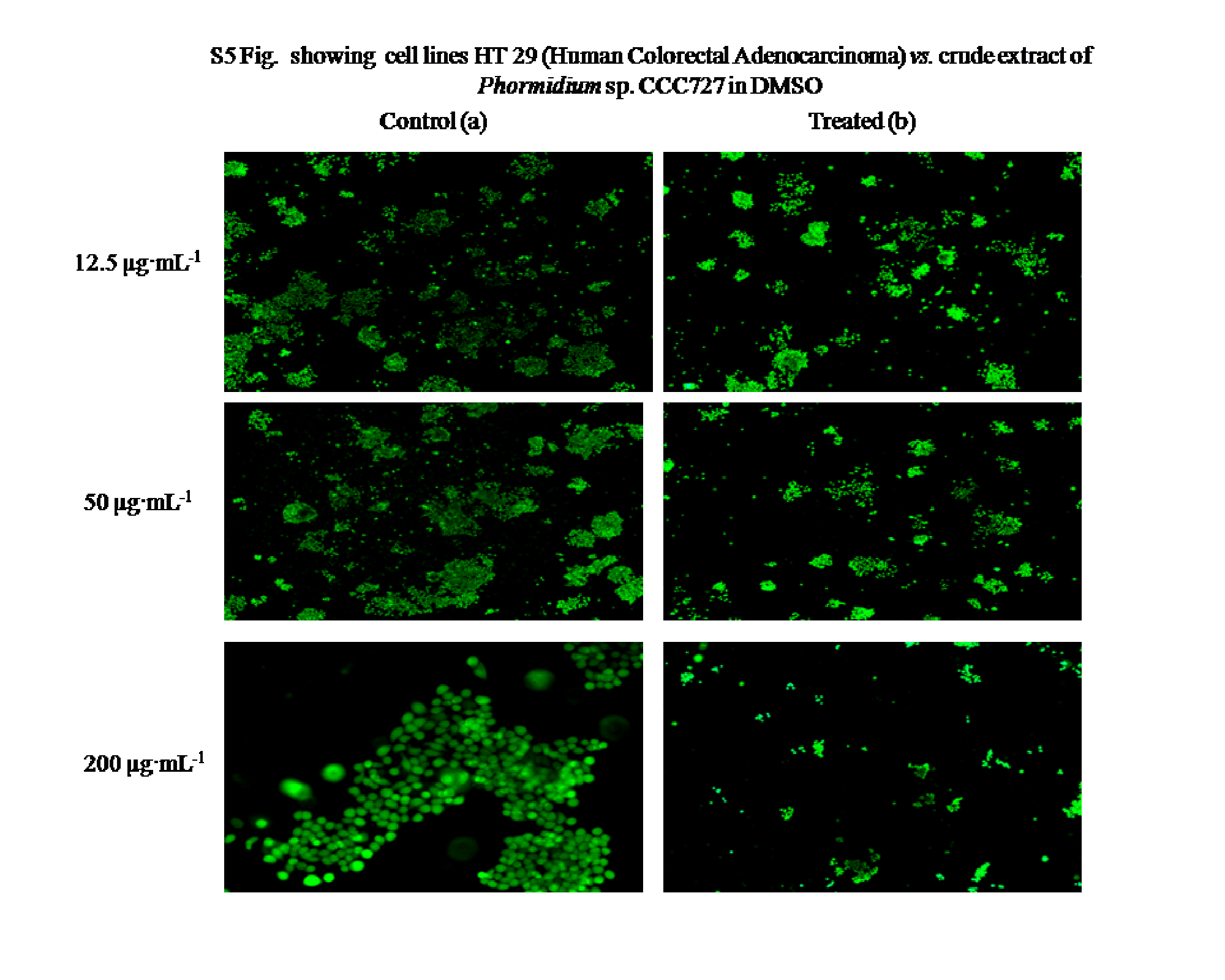

Supplement: S5 Fig — (TIF) [file pone.0136838.s005.tif]

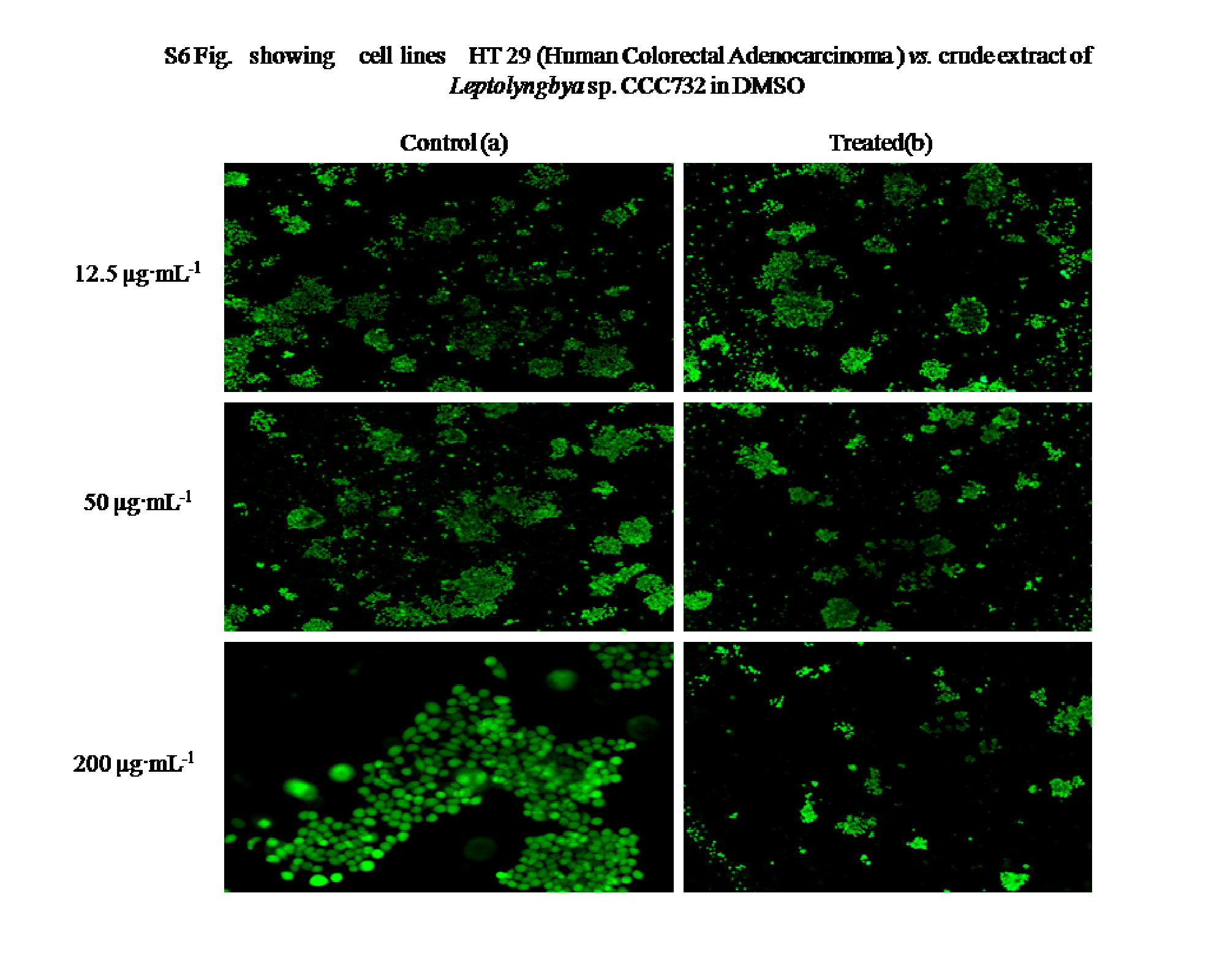

Supplement: S6 Fig — (TIF) [file pone.0136838.s006.tif]

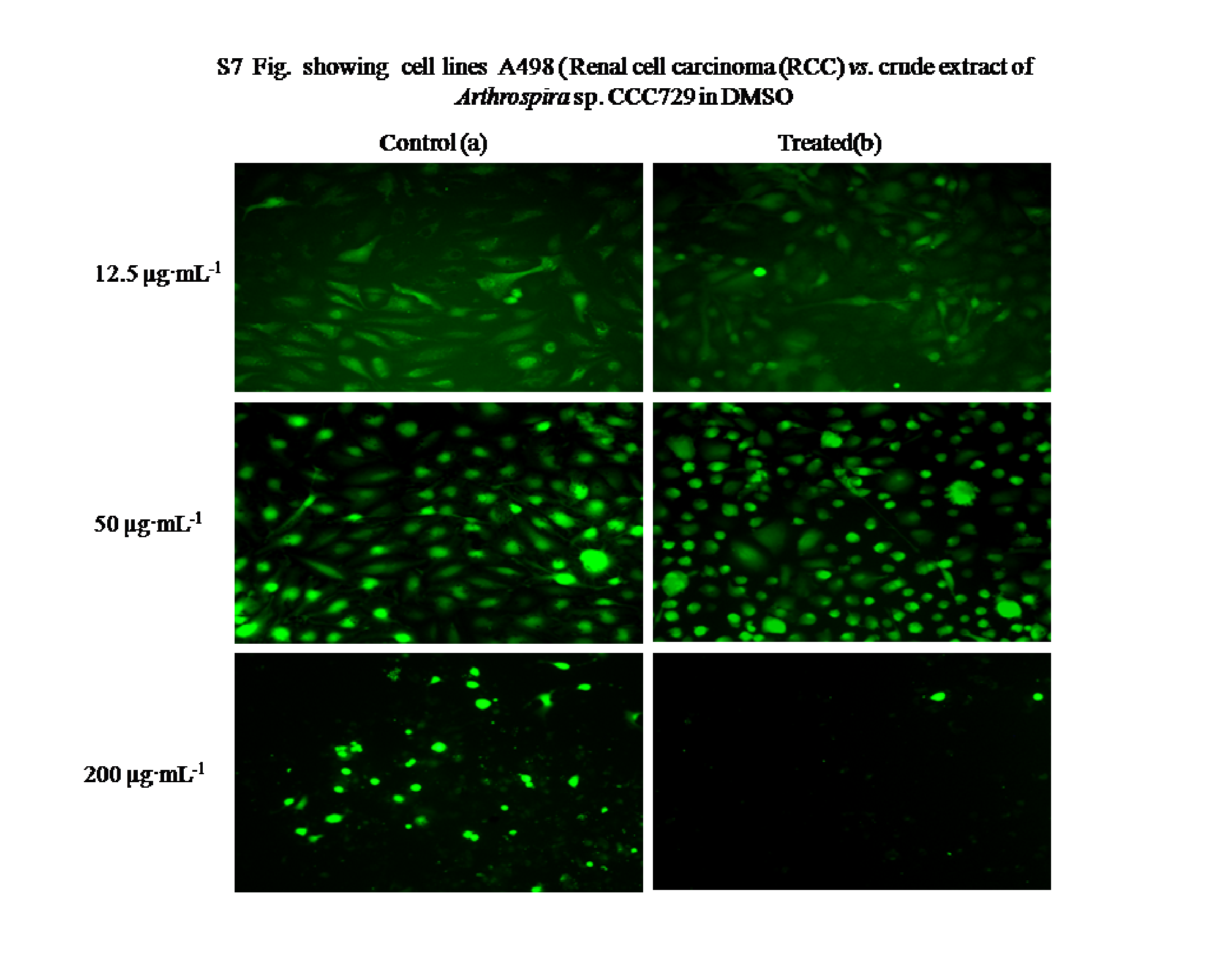

Supplement: S7 Fig — (TIF) [file pone.0136838.s007.tif]

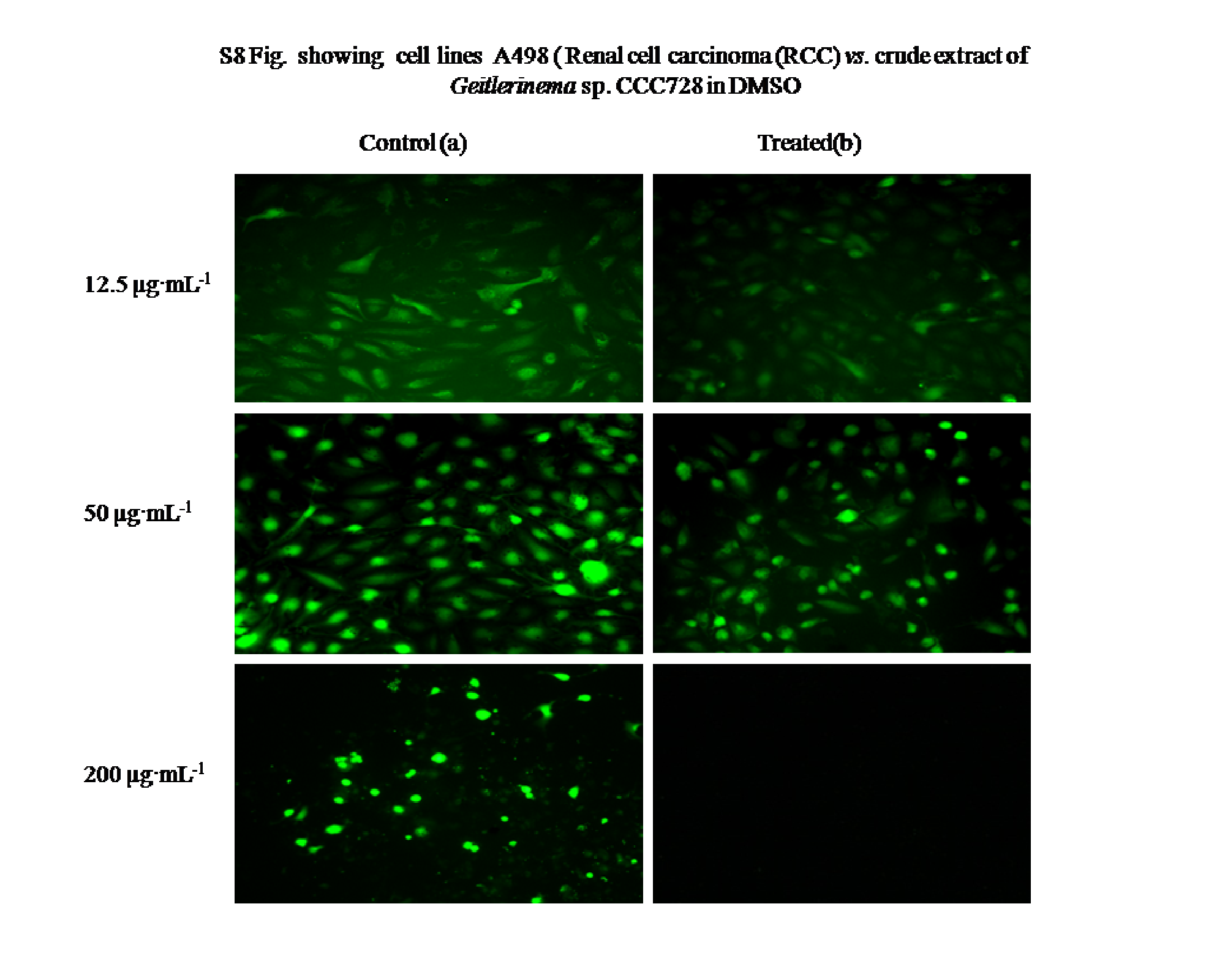

Supplement: S8 Fig — (TIF) [file pone.0136838.s008.tif]

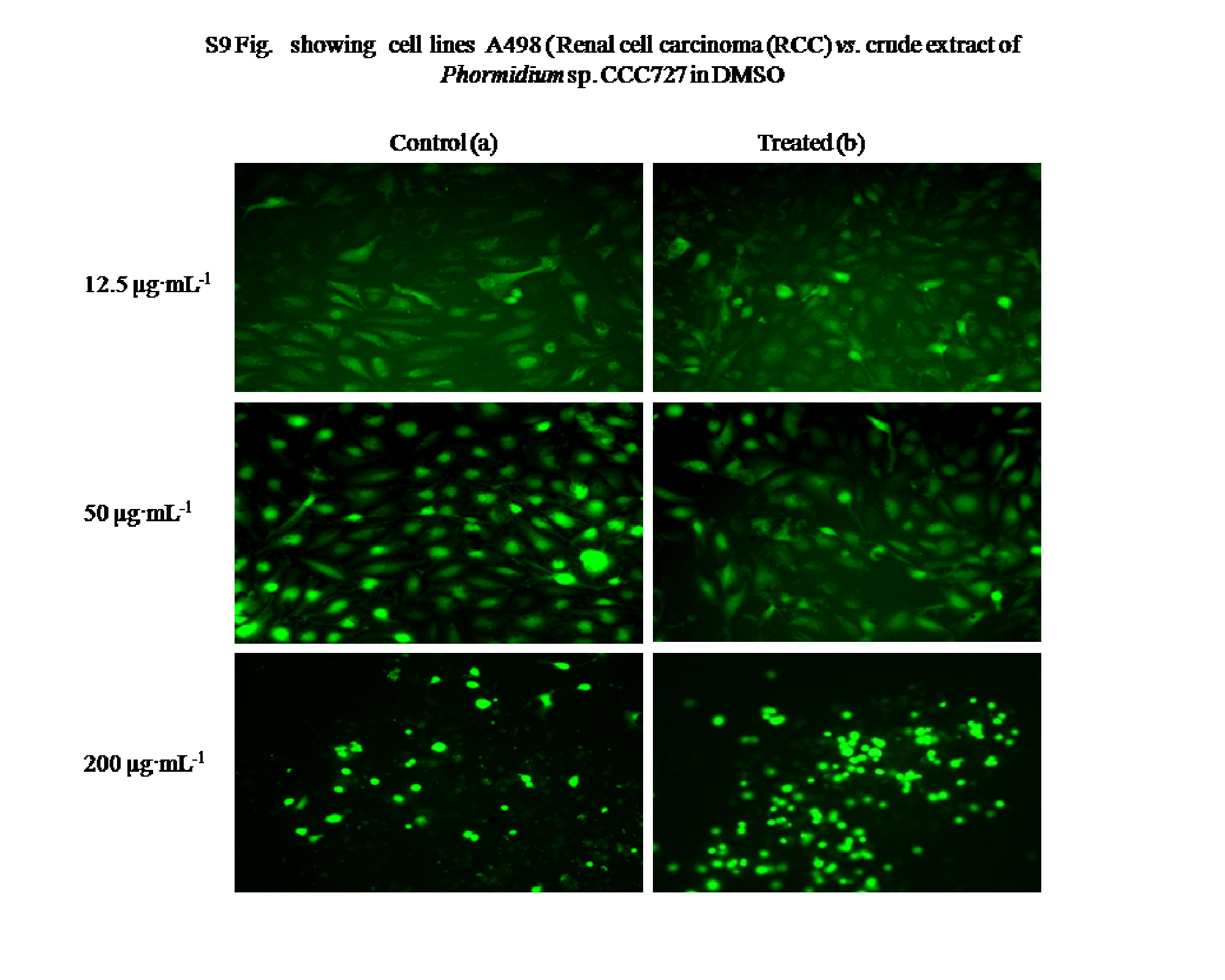

Supplement: S9 Fig — (TIF) [file pone.0136838.s009.tif]

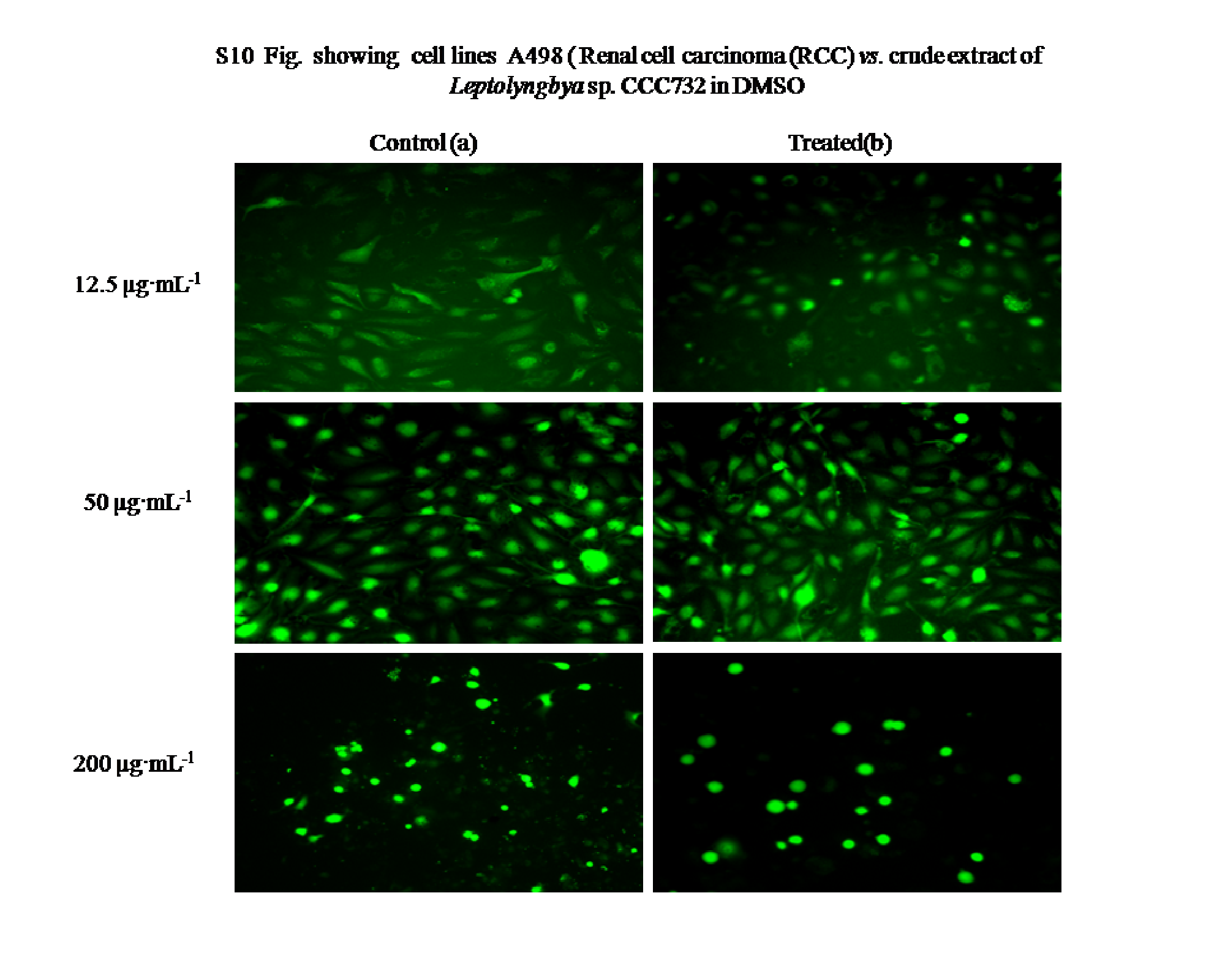

Supplement: S10 Fig — (TIF) [file pone.0136838.s010.tif]

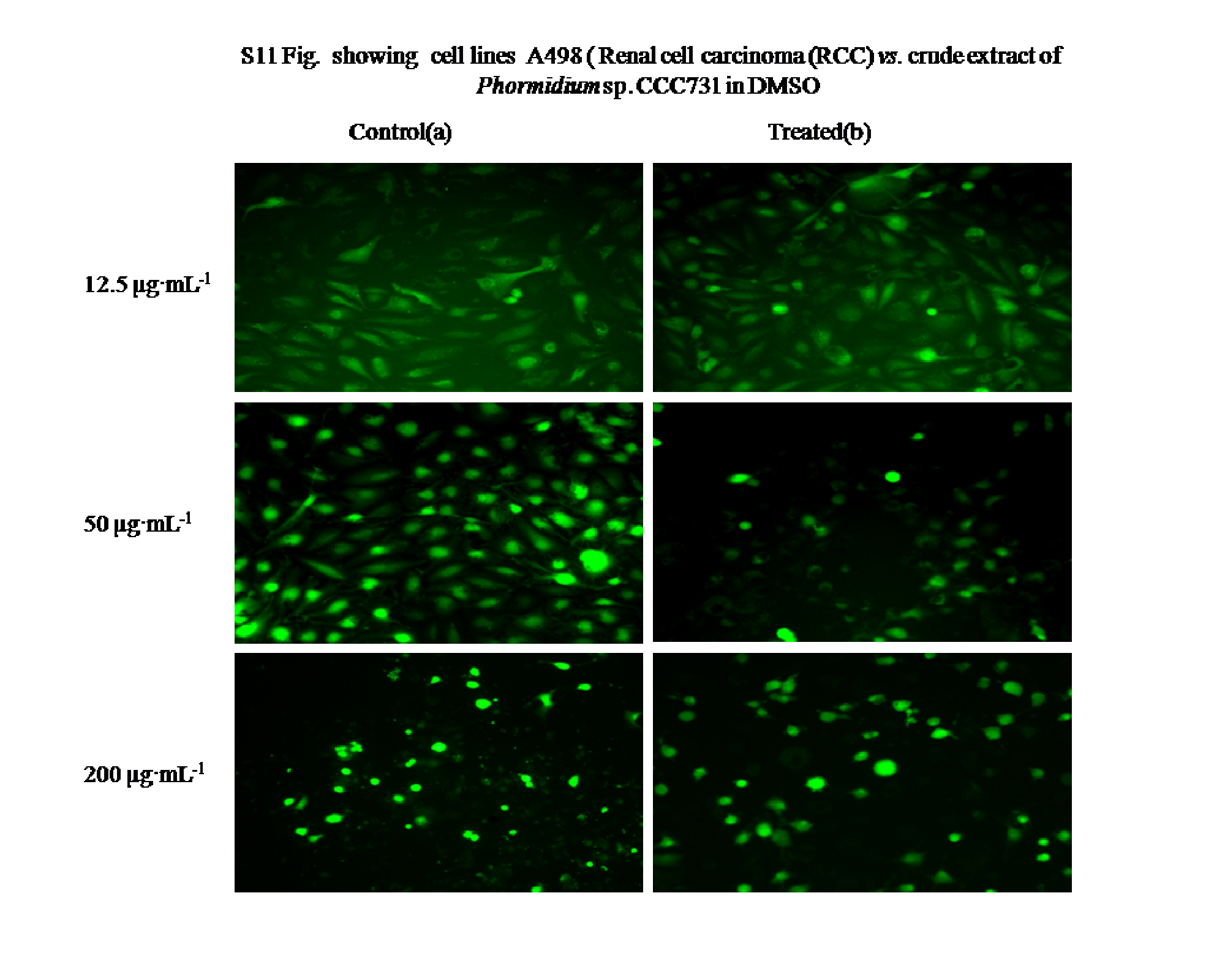

Supplement: S11 Fig — (TIF) [file pone.0136838.s011.tif]

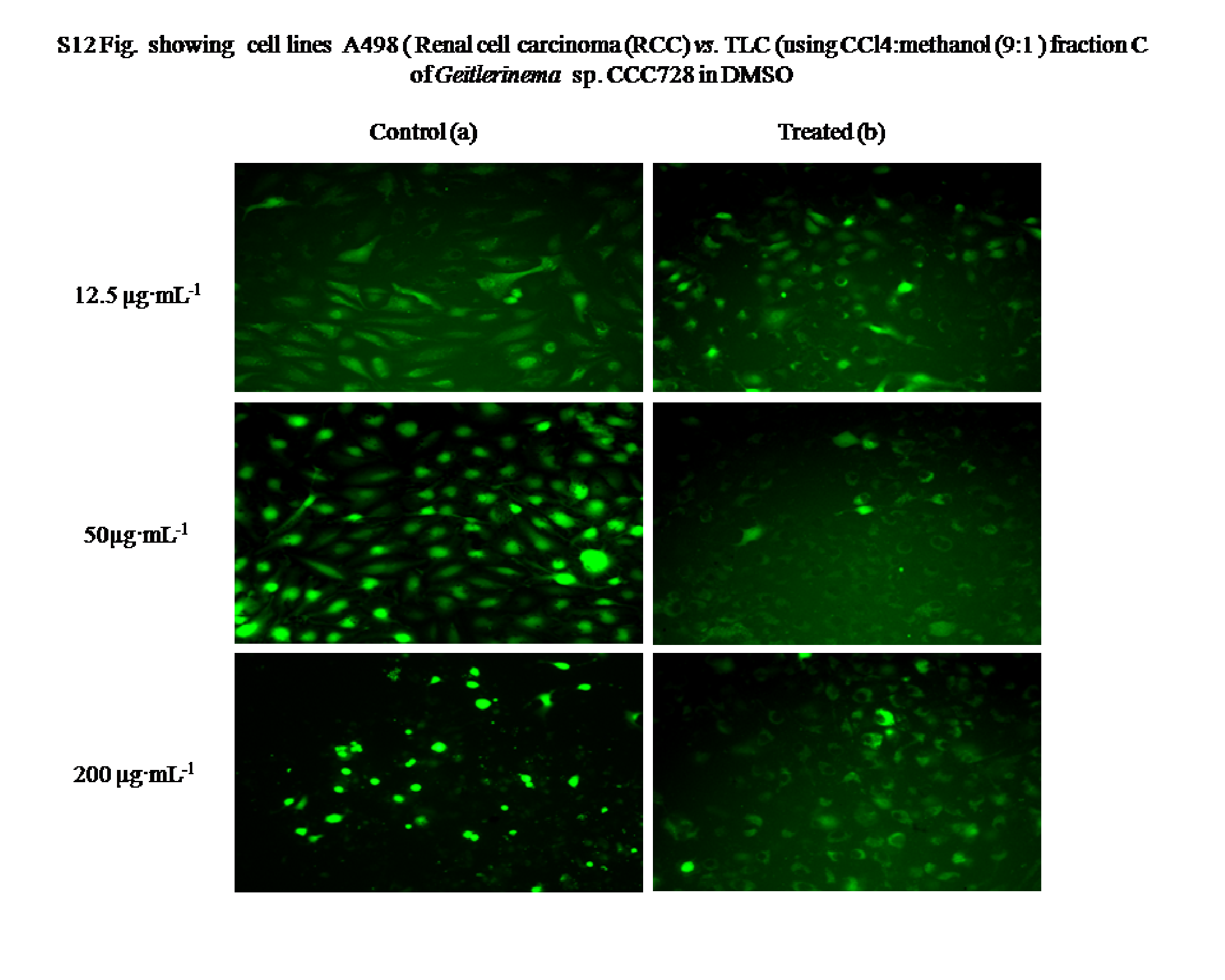

Supplement: S12 Fig — (TIF) [file pone.0136838.s012.tif]

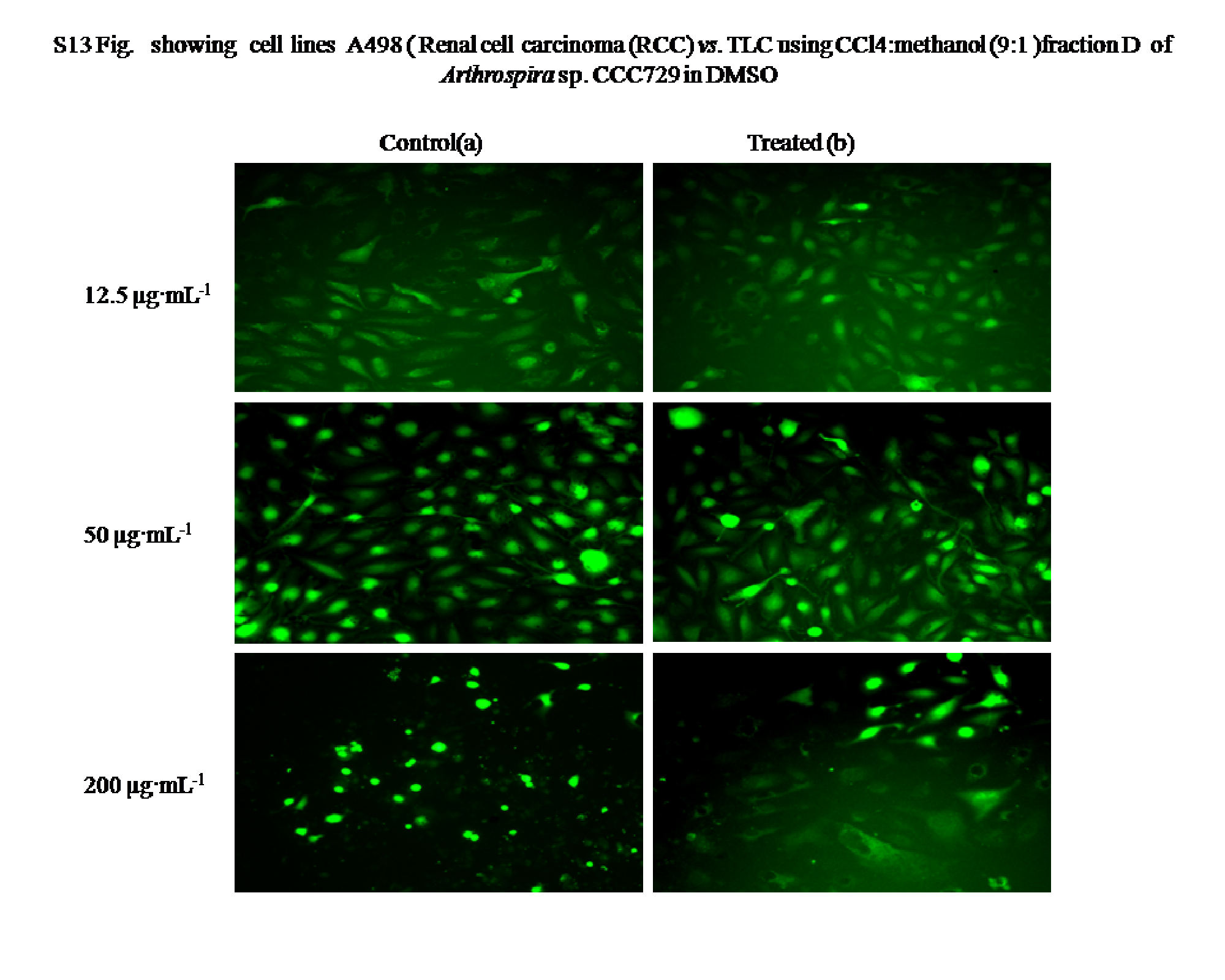

Supplement: S13 Fig — (TIF) [file pone.0136838.s013.tif]

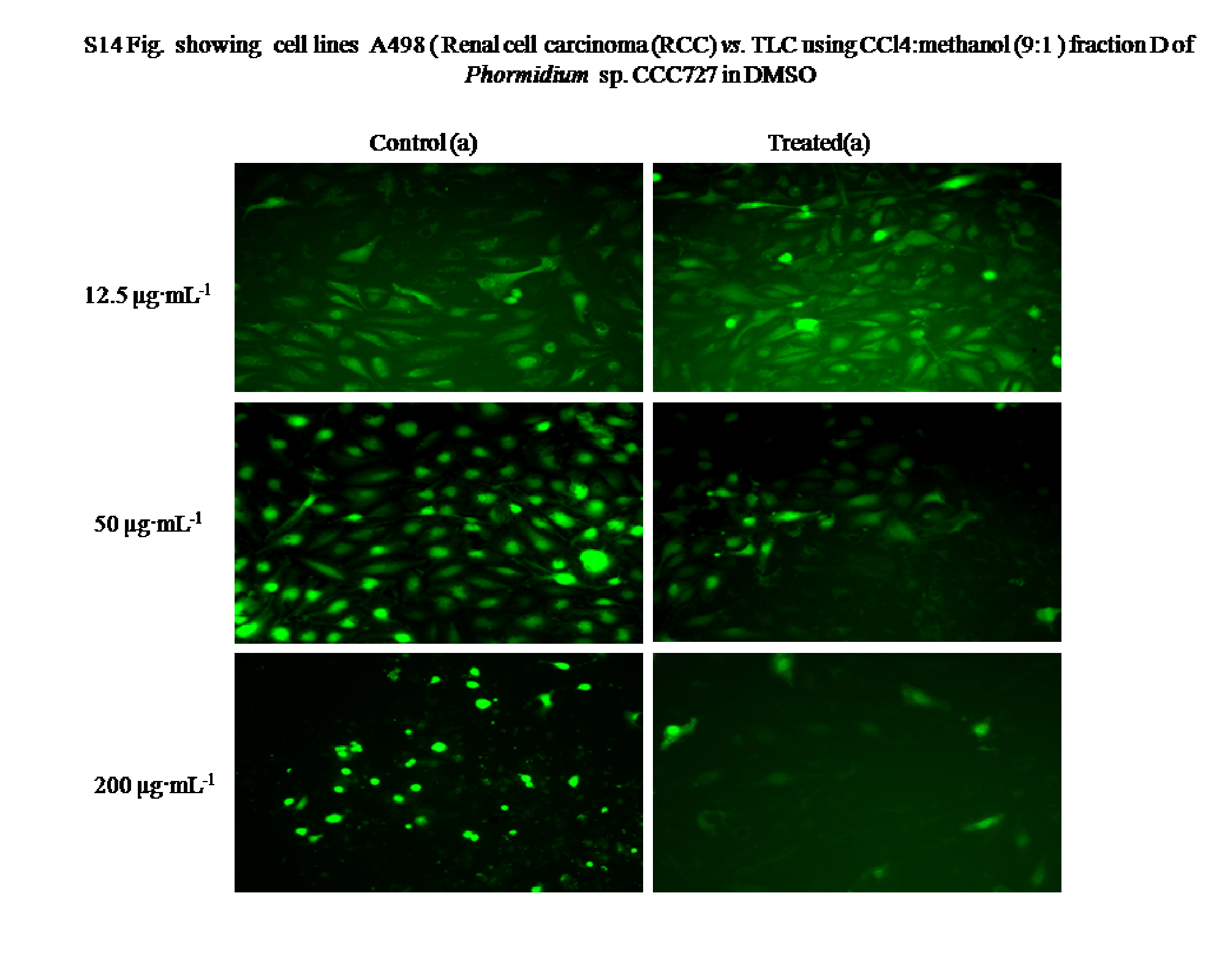

Supplement: S14 Fig — (TIF) [file pone.0136838.s014.tif]

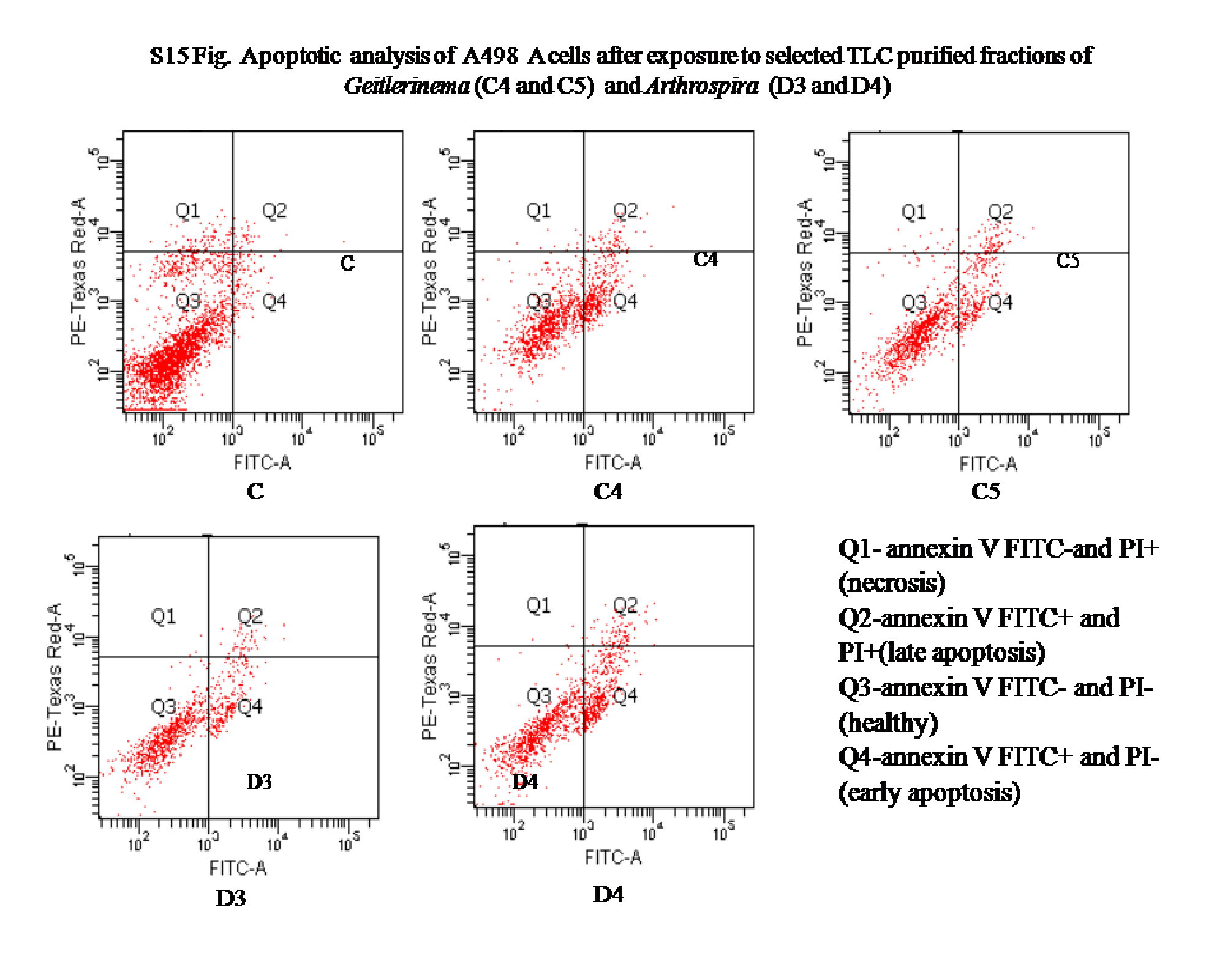

Supplement: S15 Fig — (TIF) [file pone.0136838.s015.tif]

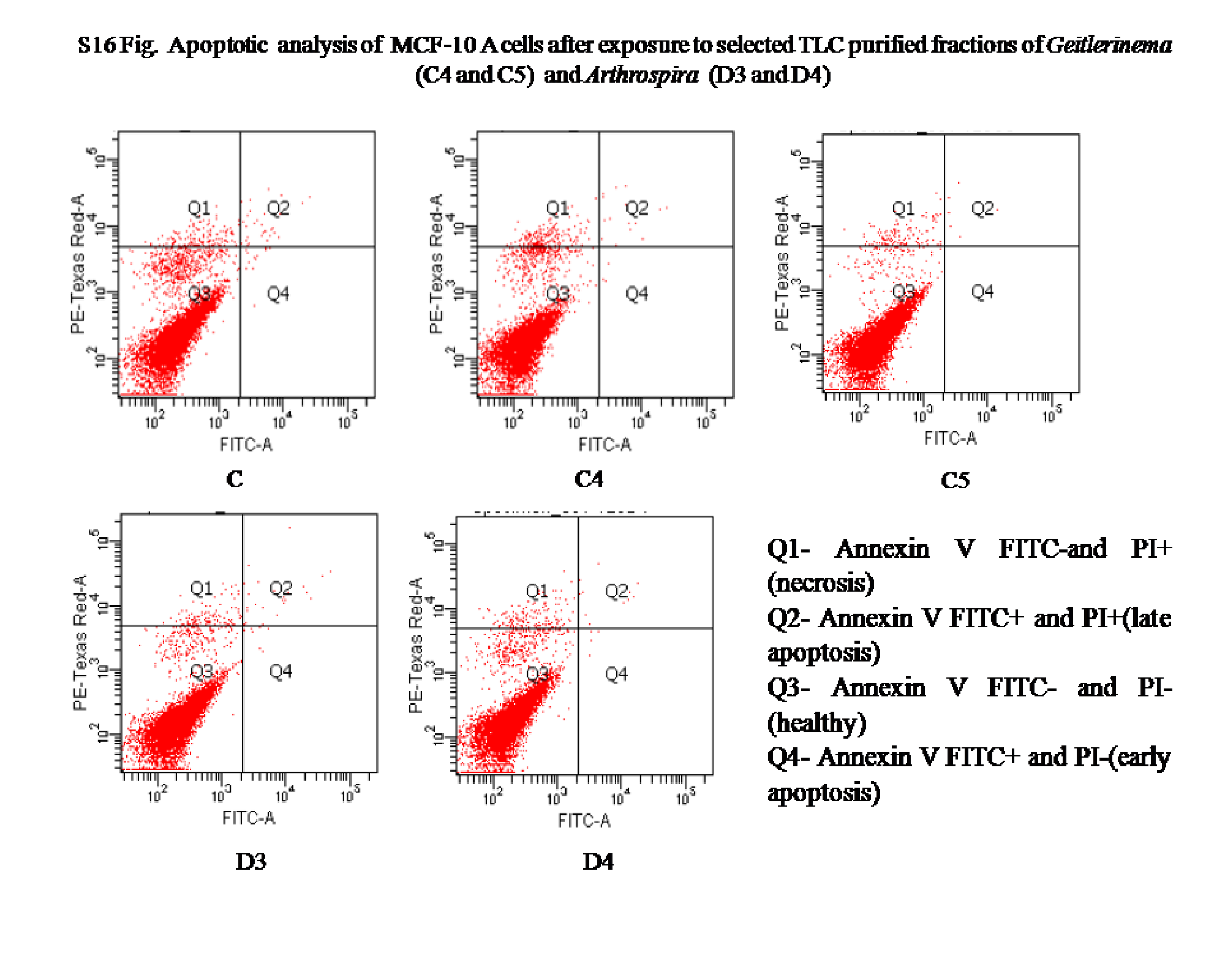

Supplement: S16 Fig — (TIF) [file pone.0136838.s016.tif]

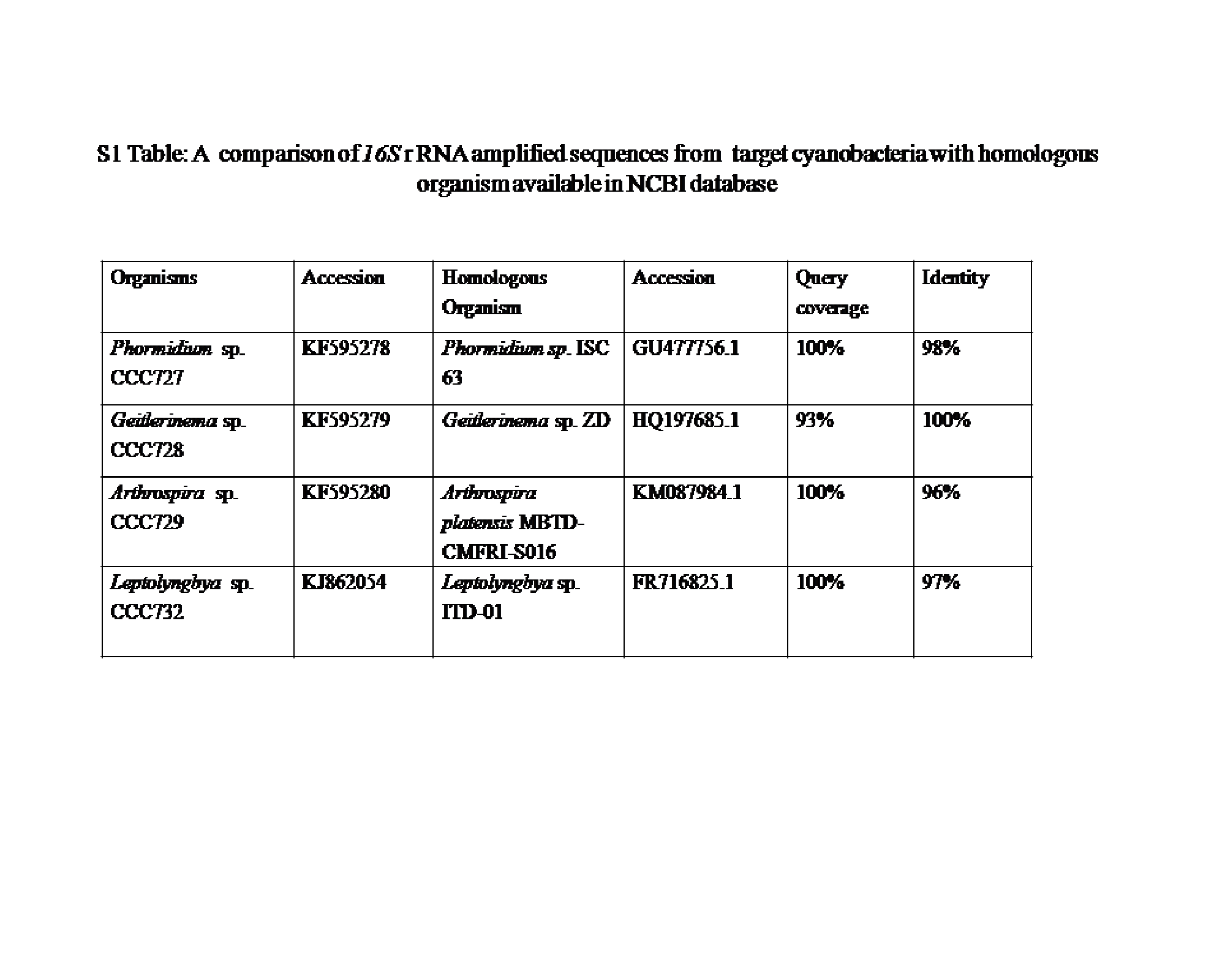

Supplement: S1 Table — (TIF) [file pone.0136838.s017.tif]
